# A mathematical model and numerical simulation for SARS-CoV-2 dynamics

**DOI:** 10.1038/s41598-023-31733-2

**Published:** 2023-03-20

**Authors:** Antonino Amoddeo

**Affiliations:** Department of Civil, Energy, Environment and Materials Engineering, Università ’Mediterranea’ di Reggio Calabria, Via Graziella 1, Feo di Vito, 89122 Reggio Calabria, Italy

**Keywords:** Computational biophysics, Applied mathematics, Immunology

## Abstract

Since its outbreak the corona virus-19 disease has been particularly aggressive for the lower respiratory tract, and lungs in particular. The dynamics of the abnormal immune response leading to lung damage with fatal outcomes is not yet fully understood. We present a mathematical model describing the dynamics of corona virus disease-19 starting from virus seeding inside the human respiratory tract, taking into account its interaction with the components of the innate immune system as classically and alternatively activated macrophages, interleukin-6 and -10. The numerical simulations have been performed for two different parameter values related to the pro-inflammatory interleukin, searching for a correlation among components dynamics during the early stage of infection, in particular pro- and anti-inflammatory polarizations of the immune response. We found that in the initial stage of infection the immune machinery is unable to stop or weaken the virus progression. Also an abnormal anti-inflammatory interleukin response is predicted, induced by the disease progression and clinically associated to tissue damages. The numerical results well reproduce experimental results found in literature.

## Introduction

The Severe Acute Respiratory Syndrome (SARS)–Corona Virus-2 (CoV-2) appeared at the end of 2019, and can be responsible for a severe inflammation of the human respiratory tract (HRT), a disease also known as Corona Virus Disease-2019 (COVID-19): it is characterized by an abnormal response of the immune system which induces the production of several inflammatory molecules going in circulation and giving rise to a so-called cytokines storm^1,2^, an event with outcome often lethal, and whose occurrence is shared with previous coronaviruses such as SARS-CoV and Middle East Respiratory Syndrome (MERS)-CoV^2,3^.

In general, upon infection, the host response begins with the detection of the pathogen associated molecular patterns (PAMP) through the pattern recognition receptors (PRR), allowing the recognition of the external pathogen and leukocytes activation, then triggering the response of the innate immunity^1,4^. This is the first source of inflammation as response of the host to the pathogen exposure. At the same time the innate immune system, which includes monocytes, macrophages, dendritic cells, mast cells, natural killer cells, neutrophils, eosinophils and basophils, represents the first barrier of the host opposing to an external thread. Since the outbreak of the COVID-19, works have put in evidence the peculiarity of the disease as well the similarities with SARS-CoV, MERS-CoV^2,5^, while previous studies and mathematical models^6–9^ can be a foundation from which to build a mathematical modelling for SARS-CoV-2 infection. In this work we are concerned with a mathematical model trying to shed some light on the COVID-19 dynamics during the early stage of the disease progression, taking into account the virus interaction with the host innate immune response. We summarize the essential biological background needed for the model presentation and discussion, while for more details as well as review papers it is very difficult to select among a wealth of excellent ones which are present in literature, then we address the interested reader to papers cited time to time and references therein.

When a pathogen intrudes a host, it is faced by monocytes, which consequently differentiate into macrophages (M) or dendritic cells (DC)^10^. Such cells, once captured the pathogen, interact with T lymphocytes, which in turn are grouped into four types of population, among which there is the family of the effector T lymphocytes: the latter includes cytotoxic T-lymphocytes or CD8^+^ T-cells, regulator T-lymphocytes or T_reg_ cells, and helper T-lymphocytes or CD4^+^ T-cells^11^. Cytokines, a broad class of peptides that includes chemokines (CK) and interleukins (IL)^1^, are important mediator of the immune response and play a significant role in cell signalling and activation of the immune response^12,13^.

Once activated as a consequence of an external threat, CD4^+^ T-cells start producing a variety of ILs^12^ that are responsible of the cell signalling, producing a broad immune response.

Macrophages differentiate from monocytes in classically activated macrophages (M1), or in alternatively activated macrophages (M2)^4,14–16^. The former are associated to a pro-inflammatory activity since they produce pro-inflammatory ILs as, for example in tumour progression^17^: tumour necrosis factor (TNF), IL-1, IL-6 and IL-12. On the other hand, M2 macrophages are responsible of an anti-inflammatory activity since they produce anti-inflammatory ILs, such as IL-10, and perform a restorative and healing function for damaged cells^18^.

ILs are molecules that can exert both pro- and anti-inflammatory functions^13,19^ and are produced not only by macrophages, but also by CD4^+^-T cells. Pro-inflammatory ILs are produced by M1 activated macrophages, and high levels of in particular IL-6 are present in severe COVID-19 patients, as well in SARS and MERS ones^2^. In a very recent review^20^ the immunoregulatory role of IL-10 in many infections has been summarized, while it has been found that IL-10 secreted in the tumour environment encourage differentiation of macrophages towards the M2 phenotype^21^.

In Lai et al.^7^ a model for the dynamics of CD8^+^-T cells during Human Immunodeficiency Virus (HIV) -1 infection at a quasi-steady state has been presented, considering contributions from both healthy and infected CD4^+^-T cells, and introducing a chemotactic contribution due to infected CD4^+^-T cells. It is found that the steady state stability depends upon attractive or repulsive nature of the chemotactic movement^22^.

In a recent work of Quirouette et al.^8^, the spread of influenza A virus (IAV) in the HRT has been modelled in terms of partial differential equations (PDEs), considering diffusion and advection terms in one spatial dimension as well the interaction of virions with both healthy and infected target cells. Models formulated in term of ordinary differential equations (ODE) have been constructed for Dengue Virus (DV) spread coupled with the immune response^23^, while a review for the viral dynamics of HIV, Hepatitis C Virus (HCV), IAV, Ebola Virus (EV), DV and Zika Virus (ZV) has been presented in Zitzmann & Kaderali^6^.

Less recently, a model describing the space–time evolution of cancer cells has been formulated in terms of PDEs, taking into account their interaction with CD8^+^-T cells, IL-27, IL-10 and interferon-γ (IFN-γ)^9^, in one spatial dimension, highlighting the role of pro- and anti-inflammatory ILs.

At the turn of the pandemic declaration of the World Health Organization, a couple of paper reported clinical results of studies carried out on COVID-19 patients in Wuhan, China, in which the plasma levels of IL-6 and IL-10^24^, and of IL-6^25^ were measured. In this frame, the present work is aimed at: (1) investigate a possible IL-6 role on M1 polarization of macrophages while they undergo chemotaxis from SARS-CoV-2 infected T cells; hence, (2) reproduce clinical observations on SARS-CoV-2 patients, linked to the dynamics of ILs and the role they play in lung damages. To build our model we were inspired by the works of Chousterman et al.^1^, Rossi et al.^2^, Channappanavar et al.^3^, while the numerical simulations were compared with clinical findings contained in Huang et al.^24^, Zhou et al.^25^, taking some points of discussion and comparison also from previous experimental works on mice^26,27^.

## The mathematical model

We consider a portion of the HRT as the domain in which an initial amount of virus (*V*) explicitly interacts with CD4^+^-T cells, from now on simply T cells (*T*), and in which the immune response involves classically (*M1*) and alternatively (*M2*) activated macrophages, IL-6 (*L*) and IL-10 (*N*). From the mass conservation we derive the model equations for the interacting species inside the considered biological domain, in terms of reaction–diffusion PDEs. We consider the spatial position identified by the vector ***x*** = (*x,y*), while *t* denotes the variable time.

We therefore introduce the relative model equations, together with a model equation accounting for infected T cells (*I*). Moreover, the parameters entering in the model are detailed and quantified in the accompanying Supplementary Information online.

Once SARS-CoV-2 intrudes the host, it binds to angiotensin-converting enzyme 2 (ACE2) receptors^25^, subsequently its presence is revealed by the toll-like receptors (TLR) from which the immune response begins with the associated inflammation^2^. ACE2 have been proven to be the receptors of SARS-CoV and SARS-CoV-2^11,28^, both targeting T cells, a trait shared with HIV-1^7,29^. Then considering only infection of such cells, the virus evolution accounts for diffusion with a coefficient *D*_*V*_, production by infected T cells at a rate *p*; the virus is cleared by immune elimination^6^ at a rate *c*:

1 $$\frac{\partial V}{{\partial t}} = \nabla \cdot \left( {D_V\nabla V} \right) + pI - cV$$

T cells diffuse at rate* D*_*T*_, undergo natural decay according to *d*_*T*_, and are infected by virus at rate *k*^6,7^; are activated by IL-6^30,31^, promoted by M1^10^, inhibited by IL-10^20,32,33^ and M2^10^ according to *ϕ*_*21*_, *ϕ*_*22*_, *ϕ*_*23*_ and *ϕ*_*24*_, respectively.

M1 produce, among others, IL-12 driving T cell polarization^34^; the presence in the airways inflammatory microenvironment of IL-4 and IL-13 induces M2 polarization of macrophages^35^, in turn expressing IL-10 which increases T cell deactivation. On the other hand T_reg_ cells, which differentiation is stimulated by IL-10, inhibit T cell and DC activation^35,36^. In the lung, T_reg_ are promoted by alveolar macrophages, with a predominant M2 phenotype^37^. Given the common features evidenced, for example, in Mannar et al.^38^ and Fardoos et al.^39^ between SARS-CoV-2 and HIV-1, along with the fact that T cells are decreased in critical COVID-19 patients^40^, we suppose that, similarly to what happens for HIV-1^7,22,41^, depending on the concentration of some CKs, in presence of the SARS-CoV-2 infection, T cells undergo chemoattraction (chemotaxis) or chemorepulsion (fugetaxis). We then assume that T cells are attracted towards infected T cells, but are repulsed by virions, allowing SARS-CoV-2 immune evasion. In Eq. (2), *χ*_*I*_ and *χ*_*V*_ are, respectively, chemotaxis and fugetaxis coefficients for T cells; *T*_*M*_, instead, represents the maximum carrying capacity of T cells population. Hence

2 $$\frac{\partial T}{{\partial t}} = \nabla \cdot \left\{ {D_T\nabla T - T\left[ {\chi _I\left( {1 - \frac{T}{T_M}} \right)\nabla I - \chi _V\left( {1 - \frac{T}{T_M}} \right)\nabla V} \right]} \right\} - d_TT - kVT + \phi _{21}L + \phi _{22}M1 - \phi _{23}N - \phi _{24}M2$$

Infected T cells are supposed to diffuse at the same rate of T cells; they are produced by the virus/T cells interaction at rate *k*, and decay at rate *δ*; it is also conceivable that the infected Tcells/IL-6 interaction can promote activation of effector T cells and deactivation of T_reg_ cells^27^, contributing to a hyper-inflammation further reducing infected T cells at rate *ϕ*_*32*_:

3 $$\frac{\partial I}{{\partial t}} = \nabla \cdot \left( {D_T\nabla I} \right) + kVT - \delta I - \phi _{ 32}IL$$

Classically activated macrophages diffuse according to *D*_*M1*_. High levels of pro-inflammatory ILs, in particular IL-6, characterize the biological milieu of the SARS-CoV-2 infection^1,2^. From one hand it can be hypothesized that, similarly to what happens in chronic wounds, where the shift from M1 to M2 phenotype is dis-regulated with a prolonged inflammatory state^14^, a persistence of M1 macrophages and their predominance over M2 phenotypes can occur. On the other side, in Hadjadj et al.^42^ it has been found that in severe and critical COVID-19 patients an excessive inflammatory response occurs with increased levels of IL-6 which can act as a chemoattractant for macrophages and consequent tissue damage. Moreover, in COVID-19 patients an extreme increase of pro-inflammatory cytokines and other factors has been observed, among which IL-6 and IFN-γ^43^. Therefore, although in the literature we have not found evidence that IL-6 induces or promotes M1 directly, due to the peculiarity of the SARS-CoV-2 infection we assume that the abnormal expression of IL-6 and IFN-γ promotes M1 at a rate *ϕ*_*41*_. Further, the inflammation resolution involves the release of anti-inflammatory ILs^18,20,21^, then M1 are inhibited by IL-10 at rate *ϕ*_*42*_.

In critical COVID-19 patients a common feature is that in lung lesions the predominant infiltrated immune cells are constituted by monocytes and macrophages^28,40^. The deeper penetration in tumour lesions of M1 macrophages with respect to M2 subtypes has been simulated in Leonard et al.^17^, Mahlbacher et al.^21^, accounted for introducing a chemotactic movement towards the lesion. The macrophage chemotaxis has been introduced also in Owen et al.^44^, studying macrophages-based therapies for drug delivery to tumour sites. M1 phenotype predominates in the early stage of inflammation and wound repair^14^, with a shift from M1 to M2 within 5–7 days post injury, being such behaviour dis-regulated in presence of chronic wounds. Given the pro-inflammatory nature of M1 macrophages, we then assume that they move chemotactically towards infected cells according to a chemotactic coefficient *χ*_*I*_:

4 $$\frac{\partial M 1}{{\partial t}} = \nabla \cdot \left[ {D_{M 1}\nabla M 1 - M1\chi _I\left( {1 - \frac{M1}{{M1_M}}} \right)\nabla I} \right] + \phi _{41}L - \phi _{42}N,$$

where *M1*_*M*_ is the maximum carrying capacity of the M1 population.

Alternatively activated macrophages M2 diffuse at rate *D*_*M2*_, and are promoted by IL-10, which induce macrophages differentiation towards M2^2,21^ at a rate *ϕ*_*51*_; the spike protein in SARS-CoV-2 is responsible of hyper production of IL-6^45^, and in general of a complicated frame of hyper-inflammation leading to the cytokines storm, but also excess of IFN- γ has been observed^43^. We assume here that the level of IL-6 is representative of inflammation and therefore reflects proportionally that of IFN- γ, which in turn can repolarize M2 macrophages towards M1 phenotype^46^. Hence, M2 are inhibited at rate *ϕ*_*52*_ according to the IL-6 increase:

5 $$\frac{\partial M2}{{\partial t}} = \nabla \cdot \left( {D_{M2}\nabla M2} \right) + \phi _{51}{\text{N}} - \phi _{52}{\text{L}}.$$

IL-6 diffuses at rate *D*_*L*_, it is produced by M1 at rate *ϕ*_*61*_^1,10,14,17^; on the other hand, IL-6 is inhibited by IL-10 at rate *ϕ*_*63*_^1,20,21,47^:

6 $$\frac{\partial L}{{\partial t}} = \nabla \cdot \left( {D_L\nabla L} \right) + \phi _{61}M1 - \phi _{63}N.$$

Finally, IL-10 diffuses at rate *D*_*N*_, is produced by M2 macrophages at rate *ϕ*_*71*_^14,20^, while it is inhibited by IL-6 at rate *ϕ*_*73*_^1^:

7 $$\frac{\partial N}{{\partial t}} = \nabla \cdot \left( {D_N\nabla N} \right) + \phi _{71}M2 - \phi _{73}L.$$

## Methods

We performed numerical simulations of the above PDE system using the COMSOL Multiphysics™ package based on the finite element method (FEM)^48^, a technique already applied to the study of biological systems^49,50^ and in presence of strong anisotropies^51^, referring to the supplementary material for details. In Eqs. (2) and (4), we set *T*_*M*_ = *T*_*r*_ and *M1*_*M*_ = *M1*_*r*_, respectively, where *T*_*r*_ and *M1*_*r*_ are reference quantities: Table 1 groups all the used parameters in dimensional and non-dimensional form, together with a short description and estimate, while once again we refer to the Supplementary Information online for details and used sources.

**Table 1:** Summary of the reference quantities and parameters used in the model.

Reference quantity	Symbol	Units	Value	
Virus	*V*_*r*_	n cm^−3^	1 × 10^7^	
T cells	*T*_*r*_	cell cm^−3^	5 × 10^6^	
T cells maximum carrying capacity	*T*_*M*_	cell cm^−3^	5 × 10^6^	
Infected T cells	*I*_*r*_	cell cm^−3^	5 × 10^6^	
M1 macrophages	*M1*_*r*_	cell cm^−3^	6.9 × 10^6^	
M1 macrophages maximum carrying capacity	*M1*_*M*_	cell cm^−3^	6.9 × 10^6^	
M2 macrophages	*M2*_*r*_	cell cm^−3^	6.9 × 10^6^	
IL-6	*L*_*r*_	cell cm^−3^	2.87 × 10^9^	
IL-10	*N*_*r*_	cell cm^−3^	2.87 × 10^9^	
Characteristic length	*l*	cm	0.1	
Characteristic diffusion coefficient	*D*	cm s^−1^	1 × 10^–6^	
Characteristic time scale	*τ*	s	1 × 10^4^	

Parameter description	Symbol	Units	Non-Dimensional Parameter	Value
Virus diffusion coefficient	*D*_*V*_	cm^2^ s^−1^	*D*_*V*_*D*^−1^	1 × 10^–2^
T cells diffusion coefficient	*D*_*T*_	cm^2^ s^−1^	*D*_*T*_*D*^−1^	5 × 10^–3^
Infected T cells diffusion coefficient	*D*_*I*_	cm^2^ s^−1^	*D*_*I*_*D*^−1^	5 × 10^–3^
M1 diffusion coefficient	*D*_*M1*_	cm^2^ s^−1^	*D*_*M1*_*D*^−1^	5 × 10^–5^
M2 diffusion coefficient	*D*_*M2*_	cm^2^ s^−1^	*D*_*M2*_*D*^−1^	5 × 10^–5^
IL-6 diffusion coefficient	*D*_*L*_	cm^2^ s^−1^	*D*_*L*_*D*^−1^	1.45 × 10^–2^
IL-10 diffusion coefficient	*D*_*N*_	cm^2^ s^−1^	*D*_*N*_*D*^−1^	1.45 × 10^–2^
Virus production coefficient	*p*	s^−1^	*pτI*_*r*_*V*_*r*_^−*1*^	1.16 × 10^–1^
Virus clearing coefficient	*c*	s^−1^	*cτ*	6.94 × 10^–2^
Infected T cells chemotactic coefficient	*χ*_*I*_	cm^5^ s^−1^ cell^−1^	*χ*_*I*_*I*_*r*_*D*^−*1*^	1 × 10^–3^
Virus fugetactic coefficient	*χ*_*V*_	cm^5^ s^−1^ cell^−1^	*χ*_*V*_*V*_*r*_*D*^−*1*^	5 × 10^–2^
T cells decay rate	*d*_*T*_	s^−1^	*d*_*T*_*τ*	2 × 10^–2^
T cells infection rate	*k*	cm^3^ s^−1^ cell^−1^	*kτV*_*r*_	7.4 × 10^–4^
T cells activation rate by IL-6	*ϕ*_*21*_	s^−1^	*ϕ*_*21*_*τL*_*r*_*T*_*r*_^−*1*^	11.5
T cells production rate by M1	*ϕ*_*22*_	s^−1^	*ϕ*_*22*_*τM1*_*r*_*T*_*r*_^−*1*^	2.3 × 10^7^
T cells inhibition rate by IL-10	*ϕ*_*23*_	s^−1^	*ϕ*_*23*_*τN*_*r*_*T*_*r*_^−*1*^	22.96
T cells inhibition rate by M2	*ϕ*_*24*_	s^−1^	*ϕ*_*24*_*τM2*_*r*_*T*_*r*_^−*1*^	9.5 × 10^4^
Infected T cells decay rate	*δ*	s^−1^	*δτ*	2 × 10^–2^
Infected T cells reduction rate by hyper-inflammation	*ϕ*_*32*_	cm^3^ s^−1^ cell^−1^	*ϕ*_*32*_*τL*_*r*_	10
M1 production rate by IL-6	*ϕ*_*41*_	s^−1^	*ϕ*_*41*_*τL*_*r*_*M1*_*r*_^−*1*^	1 × 10^–3^
M1 inhibition rate by IL-10	*ϕ*_*42*_	s^−1^	*ϕ*_*42*_*τN*_*r*_*M1*_*r*_^−*1*^	1 × 10^–4^
M2 promotion rate by IL-10	*ϕ*_*51*_	s^−1^	*ϕ*_*51*_*τN*_*r*_*M2*_*r*_^−*1*^	0.1
M2 inhibition rate by IL-6	*ϕ*_*52*_	s^−1^	*ϕ*_*52*_*τL*_*r*_*M2*_*r*_^−*1*^	1 × 10^–3^ 2 × 10^–3^
IL-6 production rate by M1	*ϕ*_*61*_	s^−1^	*ϕ*_*61*_*τM1*_*r*_*L*_*r*_^−*1*^	0.5
IL-6 inhibition rate by IL-10	*ϕ*_*63*_	s^−1^	*ϕ*_*63*_*τN*_*r*_*L*_*r*_^−*1*^	1 × 10^–2^
IL-10 production rate by M2	*ϕ*_*71*_	s^−1^	*ϕ*_*71*_*τM2*_*r*_*N*_*r*_^−*1*^	0.1
IL-10 inhibition rate by IL-6	*ϕ*_*73*_	s^−1^	*ϕ*_*73*_*τL*_*r*_*N*_*r*_^*−1*^	9.26 × 10^–5^

Details can be found in the Supplementary Information online.

Equations (1)–(7) have been integrated in a two-dimensional square domain with 1 mm^2^ area, simulating a surface of HRT, in the 0–60 non-dimensional time interval. A *t* = 0 we assume the following set of initial conditions:

8 $$\begin{gathered} V\left( {{\varvec{x}},0} \right) = {\text{exp}}\left( { - \left| {\varvec{x}} \right|^{{2}} \varepsilon^{{ - {1}}} } \right) \hfill \\ T\left( {{\varvec{x}},0} \right) = {1} - 0.{5}V({\varvec{x}},0) \hfill \\ I\left( {{\varvec{x}},0} \right) = 0.{5}V\left( {{\varvec{x}},0} \right) \hfill \\ M1\left( {{\varvec{x}}, 0} \right)  = 0.0{5} V\left( {{\varvec{x}}, 0} \right) \hfill \\ M2\left( {{\varvec{x}}, 0} \right)  = 0.{\text{1exp}}\left( { - \left| {\varvec{ x}} \right|^{{2}} \varepsilon^{{ - {1}}} } \right) \hfill \\ L\left( {{\varvec{x}},0} \right) = 0.0{5}V\left( {{\varvec{x}},0} \right) \hfill \\ N\left( {{\varvec{x}},0} \right) = 0 \hfill \\ \end{gathered}$$

In the above equations *ε* = 0.02, then we admit that the initial viral load is centred on ***x*** = (0,0) with Gaussian shape. Moreover, a fraction of T cells is initially infected, while the M1 activated macrophages are a small fraction of the viral load. At *t* = 0 the M2 activation is not yet started then we assume that a small Gaussian distribution of alternatively activated macrophages is centred at ***x*** = (0,0), IL-6 is produced according to the M1 macrophages while IL-10 is not yet produced by M2.

During the simulations the model has been tested for robustness by allowing each parameter to vary within ± 10% of the selected value, with no significant changes observed in the virus amount in the domain at *t* = 60. Instead, the numerical simulations have been performed as a function of the *ϕ*_*52*_ parameter, in order to test the biological milieu change capable to polarize macrophages towards M1 or M2 phenotype^46^, and for this reason we imposed *ϕ*_*52*_ = 1 × 10^–3^, 2 × 10^–3^.

## Results and discussion

**Figure 1:**
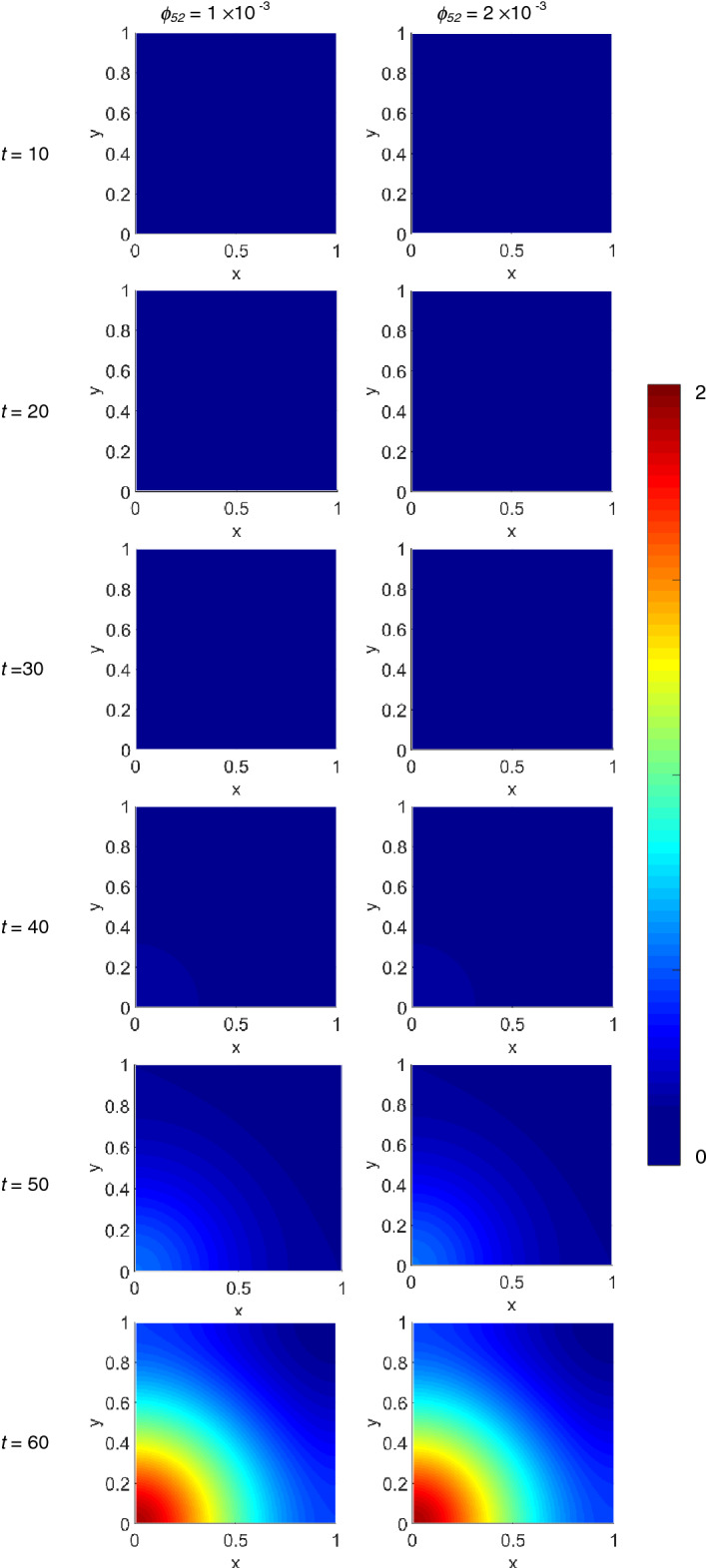
Snapshots of the virus density. The density is linearly mapped in colour scale between the blue and red colours in the [0,1] × [0,1] square domain. Starting from the top panels row, the variable evolution has been computed at *t* = 10 (~ 1.16 days), *t* = 20 (~ 2.3 days), *t* = 30 (~ 3.47 days), *t* = 40 (~ 4.63 days), *t* = 50 (~ 5.79 days) and *t* = 60 (~ 6.94 days), while the first and second column of panels refer to *ϕ*_*52*_ = 1 × 10^–3^ and *ϕ*_*52*_ = 2 × 10^–3^, respectively. All the other parameters are as in Table 1.

**Figure 2:**
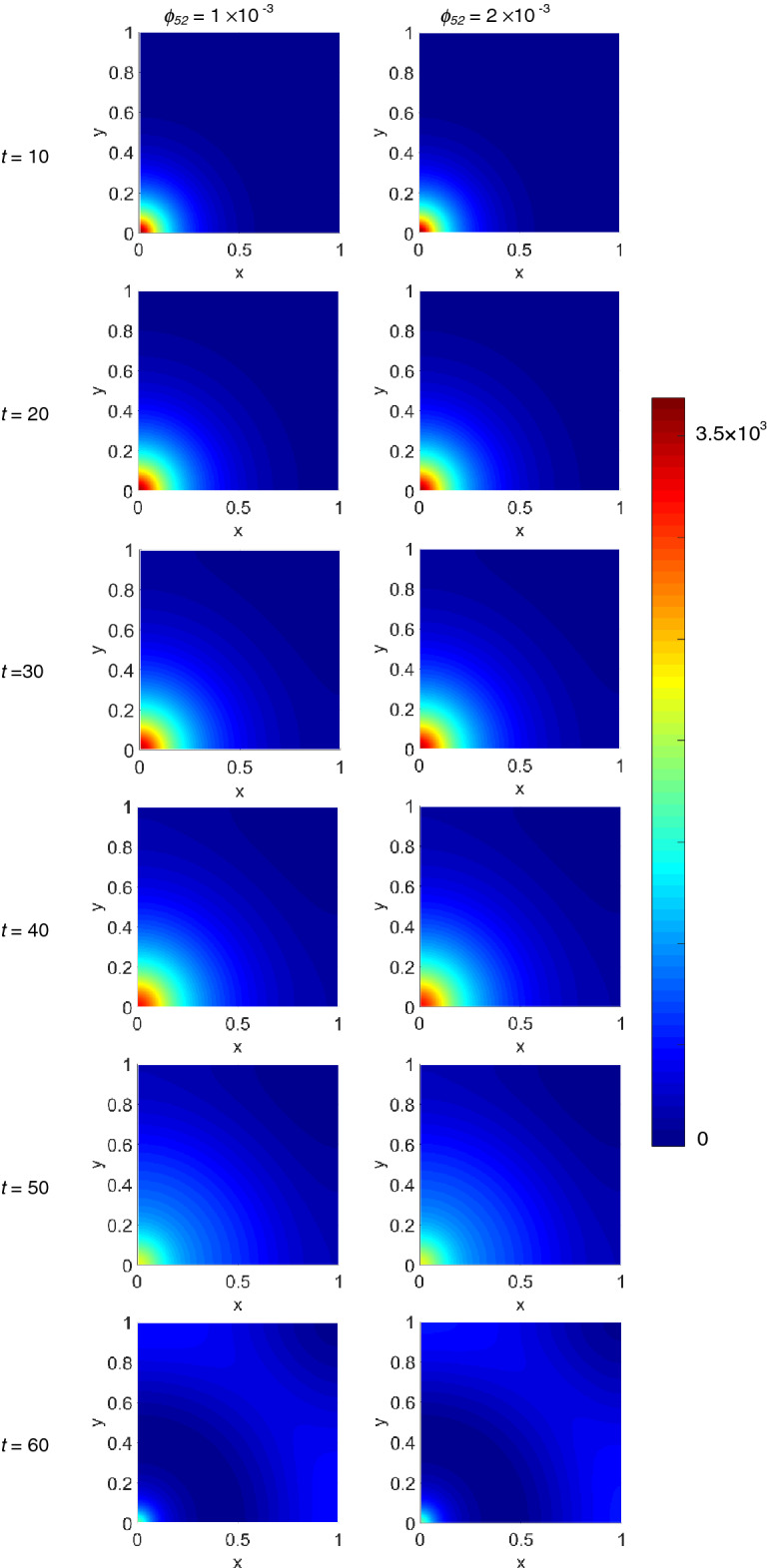
Snapshots of the T cells density. The density is linearly mapped in colour scale between the blue and red colours in the [0,1] × [0,1] square domain. Starting from the top panels row, the variable evolution has been computed at *t* = 10 (~ 1.16 days), *t* = 20 (~ 2.3 days), *t* = 30 (~ 3.47 days), *t* = 40 (~ 4.63 days), *t* = 50 (~ 5.79 days) and *t* = 60 (~ 6.94 days), while the first and second column of panels refer to *ϕ*_*52*_ = 1 × 10^–3^ and *ϕ*_*52*_ = 2 × 10^–3^, respectively. All the other parameters are as in Table 1.

We performed the numerical simulation of Eqs. (1)–(7), plotting the variables spatial distribution for selected time steps in colour scale between the blue (minimum) and the red (maximum), which at *t* = 0 obey to the initial conditions imposed with Eqs. (8). Moreover, each variable distribution has been normalized to its maximum value assumed during the time evolution, and was mapped in the [0,1] × [0,1] non-dimensional square domain. The variables evolution has been computed at *t* = 10 (~ 1.16 days), *t* = 20 (~ 2.3 days), *t* = 30 (~ 3.47 days), *t* = 40 (~ 4.63 days), *t* = 50 (~ 5.79 days) and *t* = 60 (~ 6.94 days): for each time step the results are shown in a single panels row, while the first and second column of panels refer to *ϕ*_*52*_ = 1 × 10^–3^ and *ϕ*_*52*_ = 2 × 10^–3^, respectively. We start showing in Fig. 1 the virus dynamics inside the simulated domain, and on going from *t* = 10 to *t* = 40 no features can apparently be observed, because of the damping due to the intensity normalization. At *t* = 50 the growing virus spatial distribution becomes visible as it has invaded almost half of the domain, while at *t* = 60 it has spread over the entire domain. No appreciable differences can be noted depending on the *ϕ*_*52*_ value. As expected, directly correlated to the virus dynamics is that of infected T cells, as shown in Supplementary Fig. S1 online. The dynamical evolution of the T cells is shown in Fig. 2: they progressively invade the domain as a spherical wave propagating from the origin up to *t* = 40; at *t* = 50 the primary front decreases while a secondary one starts to form, well visible at *t* = 60, insensitive to *ϕ*_*52*_ values.

The M1 density evolution is shown in Supplementary Fig. S2 online, damped and visible only close to the origin due to the intensity normalization, and for both *ϕ*_*52*_ values decreases up to *t* = 40, slightly growing from *t* = 50 on. More pronounced, instead, appears the density variation of the alternatively activated macrophages M2, which dynamical evolution is shown in Supplementary Fig. S3 online: for both *ϕ*_*52*_ values the M2 density grows progressively across the domain starting from the initial cluster at the origin, which close to the origin initially decreases up to *t* = 40, then from *t* = 50 increases again.

The IL-6 dynamics, see Supplementary Fig. S4 online, grows without appreciable differences for both *ϕ*_*52*_ values until *t* = 40, while the intensity at the origin remains almost unchanged during the propagation across the domain, then slightly decreases.

The IL-10 dynamical evolution shown in Supplementary Fig. S5 online, instead, appears more defined and seems a little faster for the lower *ϕ*_*52*_ value, and exhibits a monotonic growth.

**Figure 3:**
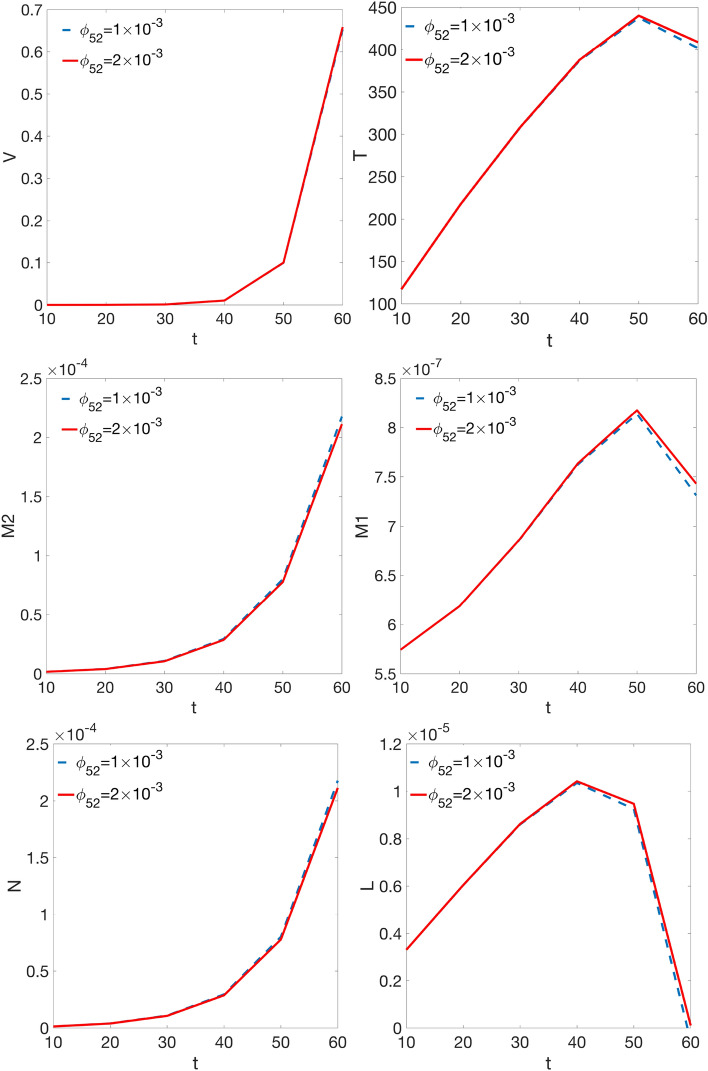
Calculated amount of the simulated species present in the domain at each time step. Points in each curve have been obtained by numerical integration over the spatial domain of the density maps shown in Figs. 1, 2 and Supplementary Figs. S2–S5 online.

The computed densities shown in Figs. 1, 2 and in Supplementary Figs. S1–S5 online, have been integrated in order to obtain, at each time step, the total amount of each species present in the domain.The results are shown in Fig. 3, except for the infected T cells which total quantity is shown in Supplementary Fig. S6 online. In each panel we plot the total amount of the species present in the domain at the simulated time steps, for *ϕ*_*52*_ = 1 × 10^–3^ (blue dashed curve), and *ϕ*_*52*_ = 2 × 10^–3^ (red continuous curve). First of all, the plots put in evidence the small differences existing between the quantities of each species present in the domain as a function of the *ϕ*_*52*_ value, sometimes not even graphically resolved. The virus amount present in the domain grows progressively as a function of time, and increases dramatically above *t* = 50, while infected T cells behave very similarly. T cells increases up to *t* = 50, then start decreasing, and very similarly behave also M1 macrophages. IL-6 grows during the time evolution but reaches a maximum at *t* = 40, and then dramatically decreases up to *t* = 60. Both M2 macrophages and IL-10 behave very similarly to the virus, and unexpectedly assume also very close values. As expected, it can be said that anti-inflammatory contributions should be promoted at low *ϕ*_*52*_ values, and vice versa for pro-inflammatory ones.

These results are consistent from a biological point of view, as M1 macrophages induce T cells activation and IL-6 production, while activated M2 macrophages induce IL-10 production.

**Figure 4:**
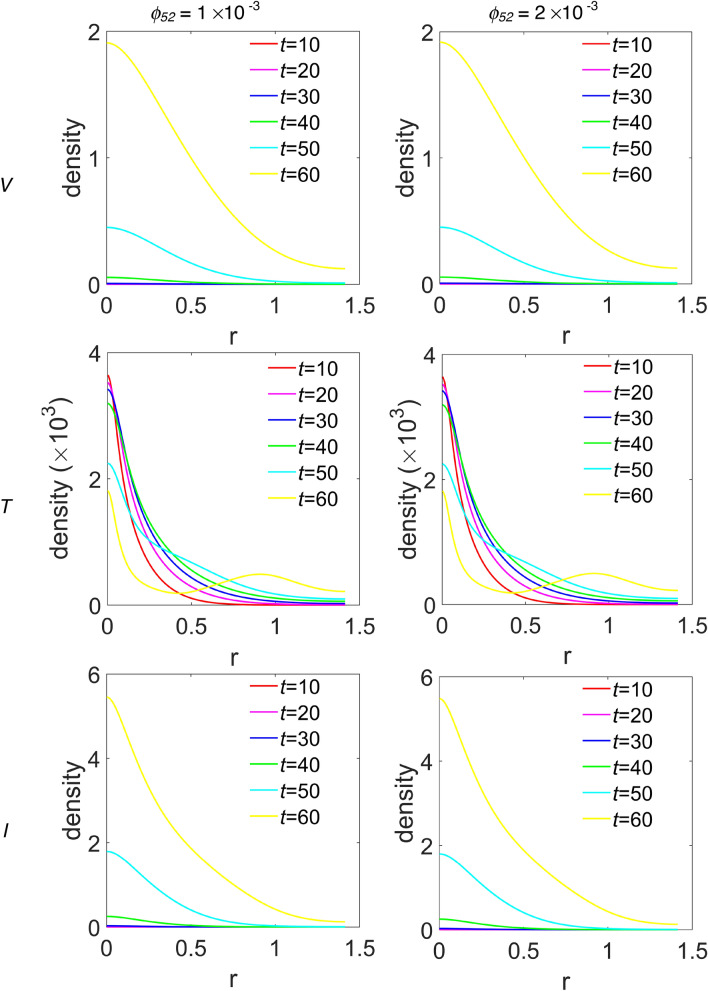
Cross-sectional densities for virions, T cells and infected T cells. Each curve refers to the density at the pertinent time step obtained along the diagonal cutline, see text for explanation. Top row refers to virions, middle row to T cells and bottom row to infected T cells; first and second panels column refer to *ϕ*_*52*_ = 1 × 10^–3^ and *ϕ*_*52*_ = 2 × 10^–3^, respectively.

In order to gain more insights on the density spatial distribution, we extract the density profiles for each species along the diagonal line cutting the 2D density maps, hence the domain, from (*x*_*1*_ = 0, *y*_*1*_ = 0) to (*x*_*2*_ = 1, *y*_*2*_ = 1), from now on referred to as the diagonal cutline, i.e., along the line splitting each density plot into two symmetric domains with respect the diagonal line. In Fig. 4 we show the obtained cross-sectional densities for virus, T cells and infected T cells. Each row of panels refers to the simulated species labelled at the beginning of the row, while first and second column of panels refer to *ϕ*_*52*_ = 1 × 10^–3^ and *ϕ*_*52*_ = 2 × 10^–3^, respectively. Inside each panel, curves referring to different time step are diversified by colour. The variable ‘r’ on the abscissa represents the distance along the diagonal cutline. The plots referring to virus and to infected T cells are very similar, as they reflect those of Fig. 1 and Supplementary Fig. S1 online, respectively, and show a growing density from *t* = 10 to *t* = 60, quite insensitive to the *ϕ*_*52*_ values. The cross-sectional density for T cells, instead, shows a predominant cells redistribution: in fact, starting from a huge peak located around the origin at *t* = 10, during the time evolution such feature decreases while broadening, and a *t* = 60 a new peak arises located along the diagonal cutline at about r = 0.9 (0.9 mm), consistent with the secondary propagation front visible in Fig. 2.

**Figure 5:**
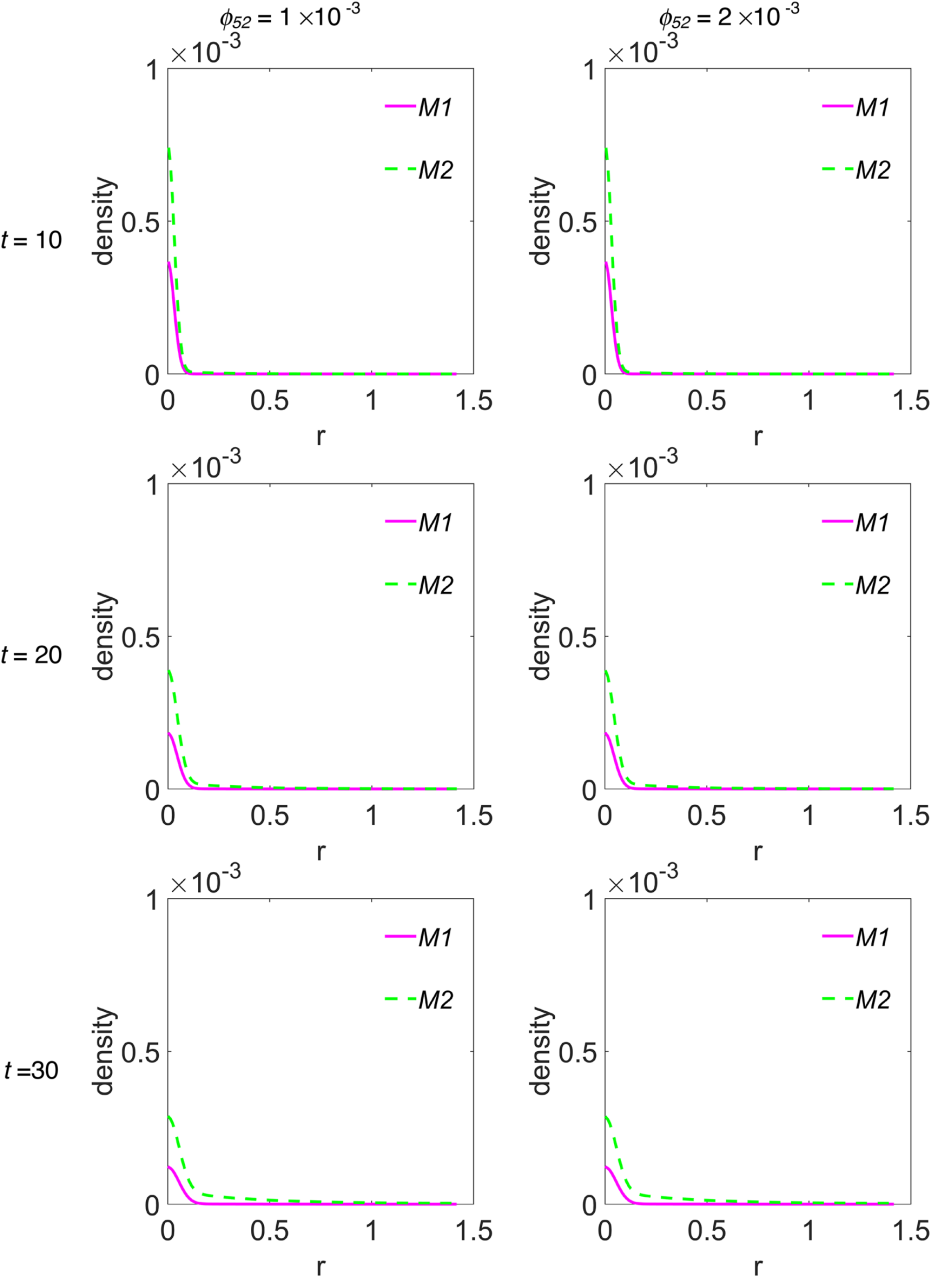

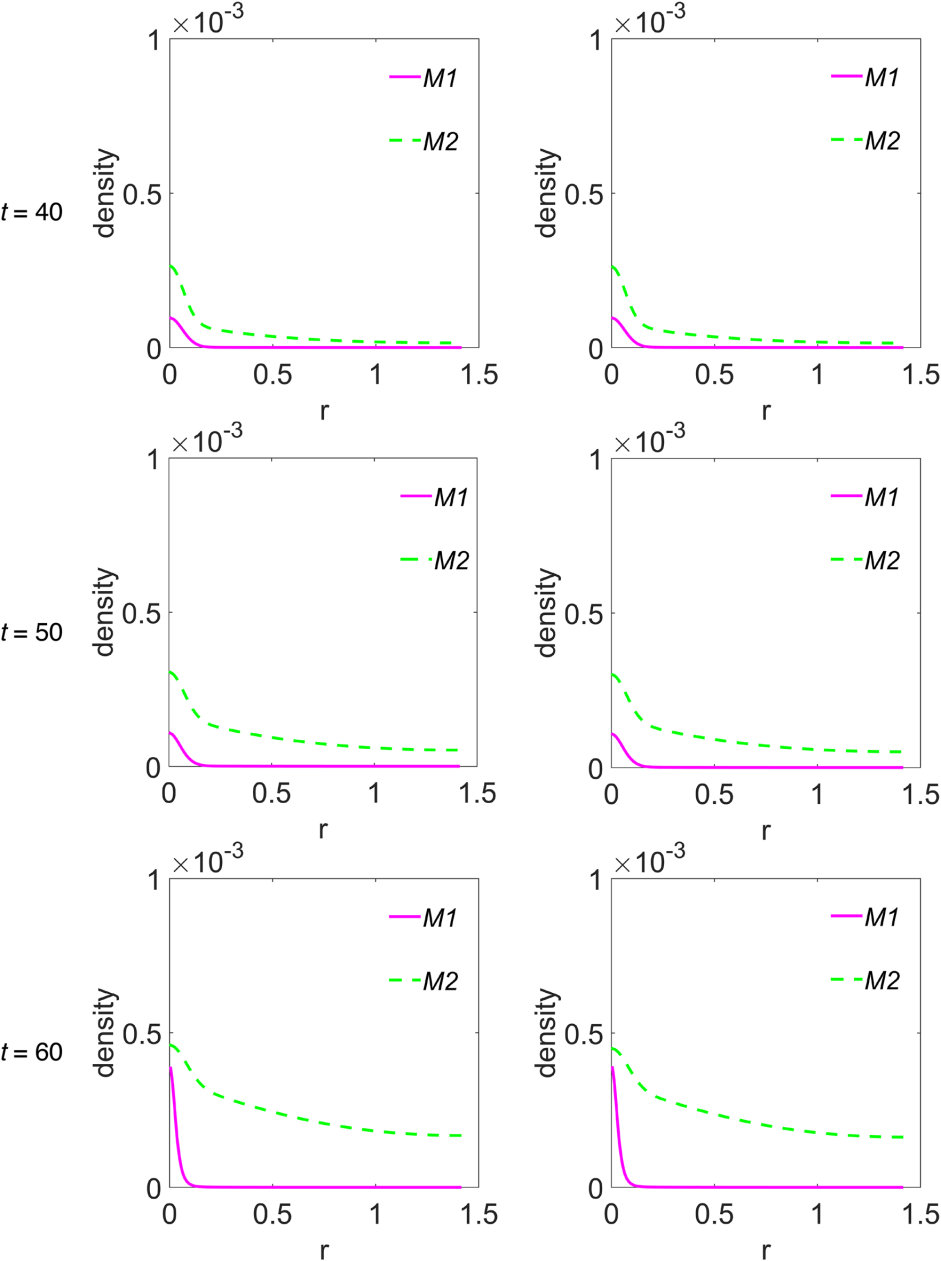
Cross-sectional densities for M1 and M2 macrophages. Each curve represents the density extracted along the diagonal cutline, see text for explanation: magenta continuous line refers to M1 macrophages, cyan dashed line to M2 macrophages; starting from the top panels row, the variables evolution have been computed at *t* = 10 (~ 1.16 days), *t* = 20 (~ 2.3 days), *t* = 30 (~ 3.47 days), *t* = 40 (~ 4.63 days), *t* = 50 (~ 5.79 days) and *t* = 60 (~ 6.94 days), while the first and second column of panels refer to *ϕ*_*52*_ = 1 × 10^–3^ and *ϕ*_*52*_ = 2 × 10^–3^, respectively. All the other parameters are as in Table 1.

**Figure 6:**
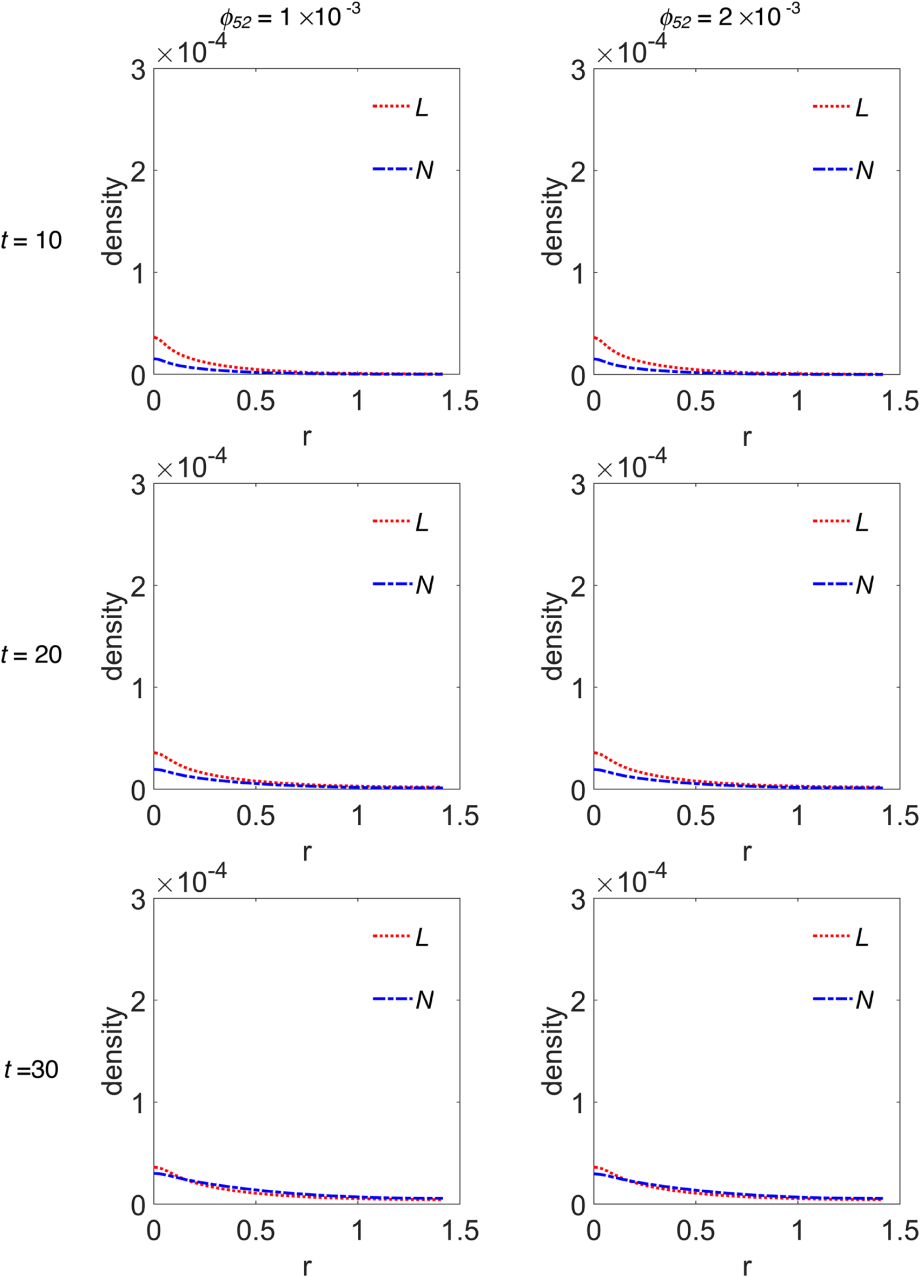

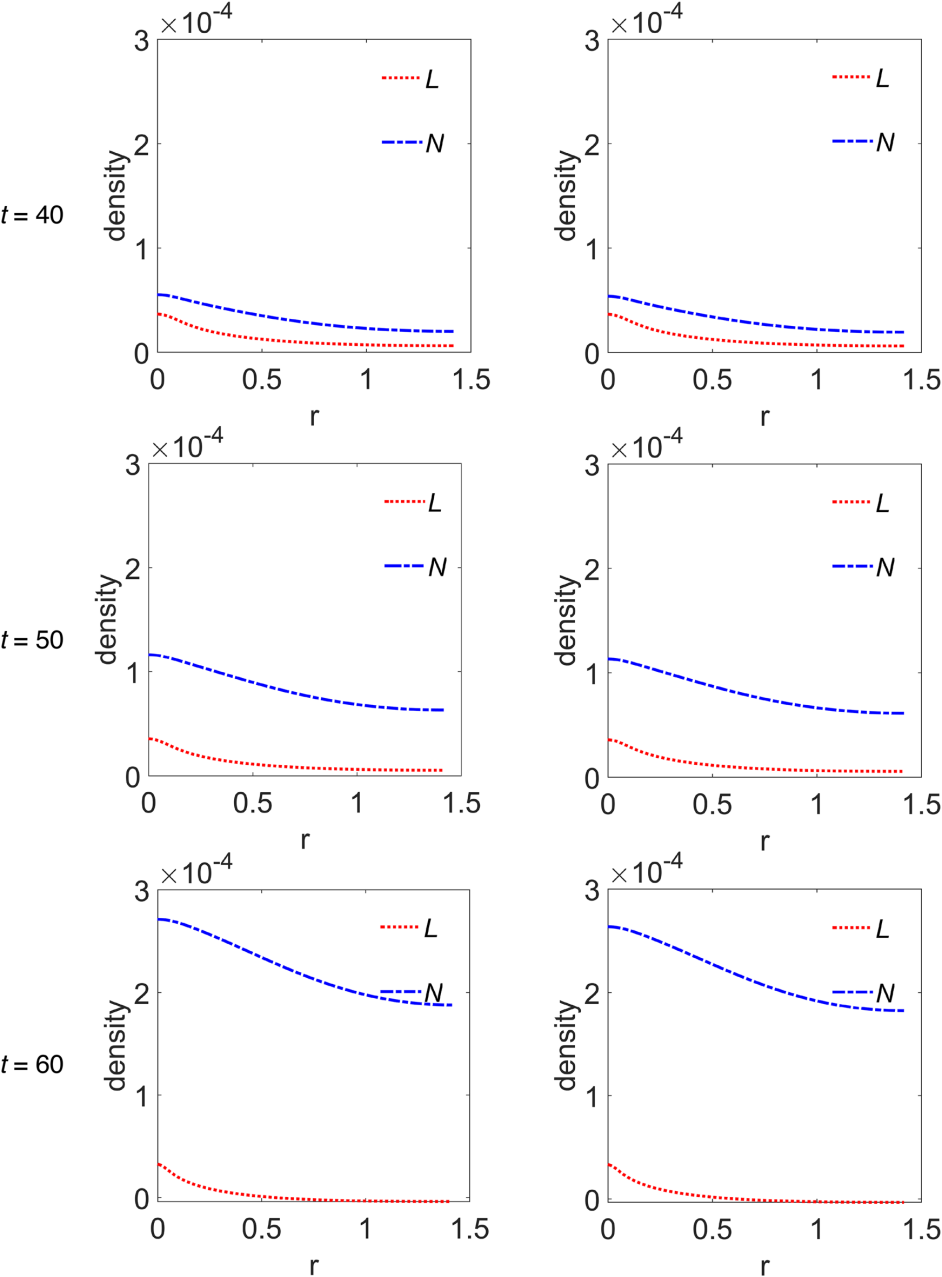
Cross-sectional densities for IL-6 and IL-10. Each curve represents the density extracted along the diagonal cutline, see text for explanation: red dotted line refers to IL-6, blue dash-dotted line to IL-10; starting from the top panels row, the variables evolution have been computed at *t* = 10 (~ 1.16 days), *t* = 20 (~ 2.3 days), *t* = 30 (~ 3.47 days), *t* = 40 (~ 4.63 days), *t* = 50 (~ 5.79 days) and *t* = 60 (~ 6.94 days), while the first and second column of panels refer to *ϕ*_*52*_ = 1 × 10^–3^ and *ϕ*_*52*_ = 2 × 10^–3^, respectively. All the other parameters are as in Table 1.

We choose to group the cross-sectional densities for macrophages and interleukins in Fig. 5 and Fig. 6, respectively, where each row of panels refers to the simulated time step, while the first and second column of panels refer to *ϕ*_*52*_ = 1 × 10^–3^ and *ϕ*_*52*_ = 2 × 10^–3^, respectively. Moreover, the variable ‘r’ has the same meaning as the previous figure. In Fig. 5, curves referring to the M1 macrophages are plotted with a magenta continuous line, while curves in cyan dashed line refer to M2 phenotype. Instead, in Fig. 6, curves referring to IL-6 are plotted with red dotted line, while those referring to IL-10 are plotted with blue dash-dotted line. The peak in the density of M1 phenotype around r = 0 decreases up to *t* = 40, then increases again from *t* = 50, indifferently for both *ϕ*_*52*_ values, see Fig. 5. The M2 density peak centred at r = 0 decreases progressively up to *t* = 40 as it widens, while increases again for *t* = 50 and *t* = 60, superimposed on a growing background indicating the penetration of the cells into the domain. Such behaviour for M2 cells seems to be more defined at *ϕ*_*52*_ = 1 × 10^–3^, as measured by the density peak at r = 0. Concerning interleukins, Fig. 6, it is evident as the IL-6 maximum intensity at r = 0 does not change on going from *t* = 10 to *t* = 60 along the diagonal cutline, but a background rises progressively up to *t* = 40, while at *t* = 60 it flattens towards zero. On the contrary, the IL-10 density grows progressively while infiltrating the domain and starting from *t* = 40 does it faster for *ϕ*_*52*_ = 1 × 10^–3^.

According to what reported in Channappanavar et al.^3^, in patients with fatal SARS and MERS the lower respiratory tract is involved, with experimental evidences of lung infiltration by monocytes/macrophages, and low counts of CD4 T cells. Our model describes such feature adequately. In fact, as can be seen in Fig. 4, during the time evolution the T cells cross-sectional density decreases in the seeding site redistributing in space with low proliferation and equally low infiltration of the simulated domain, except the weak secondary peak. At the same time, M2 macrophages, but not M1 phenotype, for r > 0 progressively infiltrate in the biological domain. Nicholls et al.^52^ and Gu et al.^53^ from one side, and Ng et al.^54^ from the other, reported lung infiltration by macrophages from autopsy samples in patients with fatal SARS and MERS, respectively. In both cases the identification of the macrophages was conducted by means of the CD68 marker, a glycoprotein highly expressed by macrophages, in particular by M2 phenotypes, which characterize an anti-inflammatory and immunosuppressive micro-environment^55^. In light of these considerations we deduce that, although our model incorporates a chemotaxis term for M1 macrophages, it describes a macrophages dynamics with M2 subtypes infiltrating the domain faster with respect to M1 subtypes. Also, the immunosuppressive and anti-inflammatory microenvironment as determined by the M2 predominance explains the low count of T cells as predicted by the model and reported by experimental findings in SARS and MERS patients^52–54^.

Du et al.^26^ studied the regulatory effects on macrophages of cannabinoid 2 receptor (CB2R) during incised skin wound healing in mice: they measured the levels of mRNA of M1 and M2 macrophages activated markers, as well the levels of pro- and anti-inflammatory cytokines, IL-6 and IL-10 among others, in mice treated with JWH133, GP1a CB2R agonist and AM630 CB2R antagonist, which were compared to the results obtained on a vehicle group. Focusing on those latter, it can be seen as M1 macrophages increase in content up to three days from the wound, then decrease; a similar behaviour is observed for M2 subtypes, which initially increase at a low rate, and then accelerate thus demonstrating the initial predominance of the M1 activated subtypes. The M2 content peaks 5 days post injury, then slightly decreases, still remaining higher than M1 subtypes. Overall, the content of both macrophages subtypes varies in the same range.

Concerning our predictions, as shown in Fig. 3, they are in part in agreement, although delayed, with the findings of Du et al.^26^, considering their reported experimental error. In fact, the M1 content grows up to 5.79 days (*t* = 50), and then decreases; on the contrary, the content of M2 macrophages increases monotonically, soon overwhelming the M1 one. Concerning the IL-6 and IL-10 content in Du et al.^26^, they reflect the behaviour of M1 and M2, respectively, and once again, their variation is contained within the same order of magnitude. Also in our simulation IL-6 and IL-10 contents reflect M1 and M2 behaviour, but IL-10 content, compared to that of IL-6, has a variation one order of magnitude larger.

Huang et al.^24^ measured the initial plasma level of, among other, IL-6 and IL-10, in COVID-19 patients from Intensive Care Unit (ICU), non-ICU patients and healthy ones, on admission to hospitalization seven days after the onset of illness. In both ICU and non-ICU patients elevated level of both interleukins, with a predominance of IL-10 in ICU patients, were found.

Further, Zhou et al.^25^ measured the dynamics of several laboratory markers including IL-6 in COVID-19 patients, survivors or with fatal outcome. Their findings, in the 4–7 days temporal range from the illness onset are in qualitative agreement with the predictions of our model, considering the reported experimental error.

Curiously, comparing the M2 and IL-10 contents in Fig. 3, they appear to be overlapping within the graphic resolution. The plots refer to the total protein amount at each time step, so we wonder if a similar correspondence can be found in the density spatial distribution during the time evolution. The answer is in Figs. 5 and 6, for M2 and IL-10 cross-sectional densities along the diagonal cutline, respectively. M2 are initially clustered in the site of infection, and during the time evolution their density decreases and broadens while infiltrating the domain, thus indicating an initial depletion at the infection site, always predominating over M1, see Fig. 5. The IL-10 level, instead, is initially lower with respect to IL-6, but from the site of infection it grows continuously while infiltrating the domain, overwhelming the IL-6 density. Then, even if the total amount of both M2 and IL-10 species are strictly comparable at each time step, their spatial distributions are not, therefore indicating different, albeit correlated, dynamics. Globally, the simulations results confirm that the initial stage of infection is characterized by a pro-inflammatory response that during the time evolution dims in favour of the anti-inflammatory reaction. While the inflammatory response is entrusted to M1 and IL-6, the anti-inflammatory reaction instead relies on M2 and IL-10, but during the initial stage of infection it is driven mainly by M2 and confined near the infection site.

Longhi et al.^27^ carried out experiments on influenza A infected mice assessing the role of IL-6 in limiting the activity of T_reg_ cells. In particular, they measured the number of CD4^+^-T cells in lung, both primary and memory (after re-infection), in wild type and IL-6 deprived mice. It can be observed as the predicted quantity of T cell shown in Fig. 3 well reproduce qualitatively the measurements for memory CD4^+^-T cells in IL-6 deprived mice reported in Longhi et al.^27^, for the corresponding time interval. It can be deduced that SARS-CoV-2 infection impairs T cells response as in an IL-6 deprived environment.

It seems that our model, accounting for SARS-CoV-2 infection, predicts some resistance to the immune machinery, as indeed the experimental results in Huang et al.^24^, and in Zhou et al.^25^ indicate. In fact, in presence of a wound healing triggered by a surgical incision^26^, the M1 behaviour is consistent with their inflammatory role during the first stage post injury, while M2 behave consistently to their anti-inflammatory and repairing role post inflammation. In our study, in the presence of a severe infection such as that caused by SARS-CoV-2, both M1 and IL-6 drive the inflammatory response, although with some delay with respect to the initial insult. The infection grows progressively in the simulated time interval, apparently unaffected by the immune response: we observe that the inflammatory response weakens, while the anti-inflammatory response is strengthened, precisely when the viral production increases. Moreover, what it seems wrong in the immune machinery is the anti-inflammatory reaction with an excess of M2 activation and IL-10 production, which could eventually lead to a damage of the respiratory tract interested by the infection, i.e., pulmonary fibrosis^26^.

We are aware that the presented model is a simplification of more complex biological mechanisms, but the qualitative agreement existing between our numerical results and clinical observations in severe COVID-19 patients is convincing of a correct schematization. It therefore constitutes a baseline from which to start including models aimed at treatments and possible therapeutic strategies.

After the manuscript submission a couple of articles appeared focusing on possible emerging epidemic threats, especially viruses coinfection, and new SARS-CoV-2 variants, indicating cutting edge research topics deserving attention. Haney et al.^56^ have conducted experiments on the coinfection of human lung cells with IAV and respiratory syncytial virus (RSV), finding the existence of hybrid virus particles (HVP) which are capable to evade anti-IAV neutralizing antibodies, thus defining in general an interaction between respiratory viruses such as SARS-CoV-2. Cao et al.^57^ have studied the evolution of the Omicron variant of SARS-CoV-2, demonstrating that mutations can evade neutralizing antibody drugs and convalescent plasma, suggesting that herd immunity and vaccine boosters could be inefficient to prevent infection from Omicron variants. We believe that mathematical modelling can give a valid contribution to such emerging research topics.

## Supplementary Information

Supplementary Information.

## Data Availability

All data generated or analysed during this study are included in this published article (and its supplementary information files).
