# Supplementary material for: A mathematical model and numerical simulation for SARS-CoV-2 dynamics
Source: Sci Rep. 2023 Mar 20;13:4575. doi: 10.1038/s41598-023-31733-2 (PMC10027279; doi:10.1038/s41598-023-31733-2)
Supplement: Supplementary file 1 — Supplementary Information. [file 41598_2023_31733_MOESM1_ESM.pdf]

SUPPLEMENTARY INFORMATION

A mathematical model and numerical simulation for SARS-CoV-2 dynamics

Antonino Amoddeo

Department of Civil, Energy, Environment and Materials Engineering, Università 'Mediterranea' di Reggio Calabria, Via Graziella 1, Feo di Vito, I-89122 Reggio Calabria, Italy

Correspondence: antonino.amoddeo@unirc.it; Tel: +39-0965-169-3299; Fax: +39-0965-169-2201.

We report here the model equations introduced in the main text.

$$\frac{\partial V}{\partial t} = \nabla \cdot \left(D_V \nabla V \right) + pI - cV \quad . \tag{1}$$

$$\frac{\partial T}{\partial t} = \nabla \cdot \left\{ D_T \nabla T - T \left[\chi_I \left(1 - \frac{T}{T_M} \right) \nabla I - \chi_V \left(1 - \frac{T}{T_M} \right) \nabla V \right] \right\} - d_T T - kVT + \phi_{21} L + \phi_{22} M_1 - \phi_{23} N - \phi_{24} M_2 \quad . \tag{2}$$

$$\frac{\partial I}{\partial t} = \nabla \cdot \left(D_T \nabla I \right) + kVT - \delta I - \phi_{32} IL \quad . \tag{3}$$

$$\frac{\partial M_1}{\partial t} = \nabla \cdot \left[D_{M_1} \nabla M_1 - M_1 \chi_I \left(1 - \frac{M_1}{M_{1_M}} \right) \nabla I \right] + \phi_{41} L - \phi_{42} N \quad , \tag{4}$$

$$\frac{\partial M_2}{\partial t} = \nabla \cdot \left(D_{M_2} \nabla M_2 \right) + \phi_{51} N - \phi_{52} L \quad . \tag{5}$$

$$\frac{\partial L}{\partial t} = \nabla \cdot \left(D_L \nabla L \right) + \phi_{61} M_1 - \phi_{63} N \quad . \tag{6}$$

$$\frac{\partial N}{\partial t} = \nabla \cdot \left(D_N \nabla N \right) + \phi_{71} M_2 - \phi_{73} L \quad . \tag{7}$$

The above system of PDEs has been integrated using the FEM and imposing zero-flux boundary

conditions on the domain boundaries, assuming that all the components stay confined within the simulated biological domain. The two-dimensional spatial domain of 1 mm^2 area has been discretized with a uniform mapped mesh with 2500 domain elements. The time discretization has been done using the implicit Euler method, and the local variable distribution was interpolated with quadratic shape functions via the Galerkin's method, see for example Amoddeo[1,2], while the FEM is a numerical technique that has been found to be efficient to solve systems of coupled non-linear PDE resulting from complex systems modelling such as fast transitions in nematic liquid crystals[3]. The PDE system has been non-dimensionalized introducing a set of reference quantities representative of typical biological values[2], used to rescale the variables and the parameters, as detailed in the following.

1. Parameters and reference quantities

The parameters were harvested from the literature, when possible, or calibrated so that the simulation results would match experimental results found in the literature. Supplementary Table S1 is divided into two sections. In the first one we report the reference quantity used for the parameters non-dimensionalization, their symbols, units, values and sources. Multiplying, respectively, x, or y, by l, and t by τ, it can recover the spatial and temporal dimensional scales, hence $\tau \times D = l^2$.

In the second section we report the parameters used: for each one, starting from the first column, we insert a short description, the used symbol, units, how to obtain the non-dimensional value, the non-dimensional value and finally the reference source, when possible.

**Reference Quantity**	**Symbol**	**Units**	**Value**	**Source**	
virus (V)	V_r	n cm^{-3}	1×10^7	Sender *et al.*[4]	
T cells (T)	T_r	cell cm^{-3}	5×10^6	assumed	
T cells maximum carrying capacity	T_M	cell cm^{-3}	5×10^6	assumed	
infected T cells (I)	I_r	cell cm^{-3}	5×10^6	assumed	
M1 macrophages ($M1$)	$M1_r$	cell cm^{-3}	6.9×10^6	Leonard *et al.*[5]	
M1 maximum carrying capacity	$M1_M$	cell cm^{-3}	6.9×10^6	Leonard *et al.*[5]	
M2 macrophages ($M2$)	$M2_r$	cell cm^{-3}	6.9×10^6	Leonard *et al.*[5]	
IL-6 (L)	L_r	cell cm^{-3}	2.87×10^9	Liao *et al.*[6], www.invivogen.com[7]	
IL-10 (N)	N_r	cell cm^{-3}	2.87×10^9	Liao *et al.*[6], www.rndsystems.com[8]	
characteristic length	l	cm	0.1	Amoddeo[1], Amoddeo[2]	
characteristic diffusion coefficient	D	$cm^2\ s^{-1}$	1×10^{-6}	Amoddeo[1], Amoddeo[2]	
characteristic time scale	τ	s	1×10^4	Amoddeo[1], Amoddeo[2]	
**Parameter Description**	**Symbol**	**Units**	**Non-Dimensional Parameter**	**Value**	**Source**

virus diffusion coefficient	D_V	cm^2 s^{-1}	$D_V D^{-1}$	1×10^{-2}	Quirouette *et al.*[9]
T cells diffusion coefficient	D_T	cm^2 s^{-1}	$D_T D^{-1}$	5×10^{-3}	Lai & Zou[10]
infected T cells diffusion coefficient	D_I	cm^2 s^{-1}	$D_I D^{-1}$	5×10^{-3}	Lai & Zou[10]
M1 diffusion coefficient	D_{M1}	cm^2 s^{-1}	$D_{M1} D^{-1}$	5×10^{-5}	Owen *et al.*[11]
M2 diffusion coefficient	D_{M2}	cm^2 s^{-1}	$D_{M2} D^{-1}$	5×10^{-5}	Owen *et al.*[11]
IL-6 diffusion coefficient	D_L	cm^2 s^{-1}	$D_L D^{-1}$	1.45×10^{-2}	Liao *et al.*[6]
IL-10 diffusion coefficient	D_N	cm^2 s^{-1}	$D_N D^{-1}$	1.45×10^{-2}	Liao *et al.*[6]
virus production coefficient	p	s^{-1}	$p\tau I_r V_r^{-1}$	1.16×10^{-1}	calibrated
virus clearing coefficient	c	s^{-1}	$c\tau$	6.94×10^{-2}	assimilated from Zitzmann & Kaderali[13]
infected T cells chemotactic coefficient	χ_I	cm^5 s^{-1} cell^{-1}	$\chi_I I_r D^{-1}$	1×10^{-3}	Lai & Zou [10]
virus fugetactic coefficient	χ_V	cm^5 s^{-1} cell^{-1}	$\chi_V V_r D^{-1}$	5×10^{-2}	Lai & Zou [10]
T cells decay rate	d_T	s^{-1}	$d_T\tau$	2×10^{-2}	estimated from Liao *et al.*[6]
T cells infection rate	k	cm^3 s^{-1} cell^{-1}	$k\tau V_r$	7.4×10^{-4}	calibrated
T cells activation rate by L	ϕ_{21}	s^{-1}	$\phi_{21}\tau L_r T_r^{-1}$	11.5	assimilated from Liao *et al.*[6]
T cells production rate by M1	ϕ_{22}	s^{-1}	$\phi_{22}\tau M1_r T_r^{-1}$	2.3×10^{7}	calibrated
T cells inhibition rate by N	ϕ_{23}	s^{-1}	$\phi_{23}\tau N_r T_r^{-1}$	22.96	assimilated from Liao *et al.*[6]
T cells inhibition rate by M2	ϕ_{24}	s^{-1}	$\phi_{24}\tau M2_r T_r^{-1}$	9.5×10^{4}	calibrated
infected T cells decay rate	δ	s^{-1}	$\delta\tau$	2×10^{-2}	estimated from Lai & Zou[10], Zitzmann & Kaderali[13]
infected T cells reduction rate by hyper-inflammation	ϕ_{32}	cm^3 s^{-1} cell^{-1}	$\phi_{32}\tau L_r$	10	calibrated
M1 production rate by IL-6	ϕ_{41}	s^{-1}	$\phi_{41}\tau L_r M1_r^{-1}$	1×10^{-3}	estimated
M1 inhibition rate by IL-10	ϕ_{42}	s^{-1}	$\phi_{42}\tau N_r M1_r^{-1}$	1×10^{-4}	estimated
M2 promotion rate by IL-10	ϕ_{51}	s^{-1}	$\phi_{51}\tau N_r M2_r^{-1}$	0.1	estimated
M2 inhibition rate by IL-6	ϕ_{52}	s^{-1}	$\phi_{52}\tau L_r M2_r^{-1}$	1×10^{-3}	estimated
				2×10^{-3}	estimated
IL-6 production rate by M1	ϕ_{61}	s^{-1}	$\phi_{61}\tau M1_r L_r^{-1}$	0.5	estimated
IL-6 inhibition rate by IL-10	ϕ_{63}	s^{-1}	$\phi_{63}\tau N_r L_r^{-1}$	1×10^{-2}	estimated
IL-10 production rate by M2	ϕ_{71}	s^{-1}	$\phi_{71}\tau M2_r N_r^{-1}$	0.1	estimated
IL-10 inhibition rate by IL-6	ϕ_{73}	s^{-1}	$\phi_{73}\tau L_r N_r^{-1}$	9.26×10^{-5}	assimilated from Nickaeen *et al.*[18]

Supplementary Table S1. Summary of the reference quantities and parameters used in the model.

In the following we detail the parameters values or, in the absence, their estimation/assumption or assimilation to other parameters.

1.1. Reference Quantities

Reference quantity for viral load – V_r. Sender *et al.*[4] have estimated the number of virions in humans extrapolating the data collected from experiments on rhesus macaques 2-4 days after SARS-CoV-2 inoculation. In lungs, they estimate a number of 10^6-10^8 n g^{-1}. Using then a lung density ρ=1g/1mL, we extrapolate a range 10^6-10^8 n cm^{-3}, while we assume $V_r = 10^7$ n cm^{-3}.

Reference quantity for CD4$^+$-T cells – T_r. It is commonly recognized that in adults and adolescents the number of such cells in the blood can vary in the range 500-1200 cell mm^{-3}; we then assume as reference the value 5×10^6 cell cm^{-3}.

CD4$^+$-T cells maximum carrying capacity – T_M. Set equal to T_r.

Reference quantity for infected CD4$^+$-T cells – I_r. It is considered the same as T_r.

Reference quantity for M1 activated macrophages – $M1_r$. Leonard *et al.*[5] assumed the number of macrophages recruited by a tumour lesion is about 6.9×10^6 cell cm^{-3}. Hence, in the absence of measured data for SARS-CoV-2 infection, we assume the same value.

M1 macrophages maximum carrying capacity – $M1_M$. Set equal to $M1_r$.

Reference quantity for M2 activated macrophages – $M2_r$. It is considered the same as $M1_r$.

Reference quantity for IL-6 – L_r. The paper of Liao *et al.*[6] reports on the mathematical modelling of the anti-tumour response induced by interleukin-27 (IL-27), involving also CD8$^+$ -T cells, IL-10 and IFN-γ, where as reference quantity for IL-27, IL-10 and IFN-γ it is assumed the value of 10^2 pg cm^{-3}. Due to its pro-inflammatory role, we assimilate IL-6 to IFN-γ, assuming for what concerns us a reference quantity for IL-6 = 10^2 pg cm^{-3}. Being the molecular mass of IL-6, $M_{IL\text{-}6} \approx 21$ kDa[7], the Avogadro number $N_A = 6.022\times10^{23}$, then the number of IL-6 cells contained in 10^2 pg is given by

$$\frac{mass\ (g)}{molecular\ mass\ (Da)} \times N_A = \frac{10^{-10}}{21\times10^3}\frac{g}{Da} \times 6.022 \times 10^{23} \approx 2.87 \times 10^9.$$

We then assume $L_r = 2.87 \times 10^9$ cell cm^{-3}.

Reference quantity for IL-10 – N_r. Still from the previous case, a reference quantity for IL-10 it is assumed[6] as 10^2 pg cm^{-3}. Being the molecular mass of IL-10, $M_{IL\text{-}10} \approx$ 17-21 kDa[8], choosing the higher value for convenience, as for L_r we obtain $N_r = 2.87 \times 10^9$ cell cm^{-3}.

Characteristic length – l, characteristic diffusion coefficient – D, characteristic time scale – τ.

Our simulations are modelled in a square domain with side 1 mm, which is a typical length for biological phenomena, then $l = 0.1$ cm.

A representative chemical diffusion coefficient is $D = 1 \times 10^{-6}$ cm^2 s^{-1}.

A typical time scale is then obtained by $l^2 D^{-1} = 1 \times 10^4$ s. See Amoddeo[1,2].

1.2. Parameters

Virus diffusion coefficient – D_V. We choose this parameter according to Quirouette *et al.*[9] for influenza A dynamics in human respiratory tract (HRT): $D_V = 1 \times 10^{-12}$ m^2 s^{-1} = 1×10^{-8} cm^2 s^{-1}.

$CD4^+$-T cell diffusion coefficient – D_T. In Lai *et al.*[10] non-dimensional diffusion coefficients for $CD4^+$-T cell of 5×10^{-3} and 1×10^{-1} are reported, in the context of a mathematical model for HIV-1 infection dynamics with $CD8^+$-T cells response. On the other hand, in Liao *et al.*[6] a diffusion coefficient of 5×10^{-11} cm^2 s^{-1} for $CD8^+$-T cells has been assumed. Hence, we adopt a non-dimensional coefficient $D_T = 5 \times 10^{-3}$.

Infected $CD4^+$-T cell diffusion coefficient – D_I. It is considered the same as D_T.

M1 macrophages diffusion coefficient – D_{M1}. We adopt the value reported in Owen *et al.*[11] for macrophages infiltration in tumours, then we assume $D_{M1} = 5 \times 10^{-11}$ cm^2 s^{-1}.

M2 macrophages diffusion coefficient – D_{M2}. It is considered the same as D_{M1}.

IL-6 diffusion coefficient – D_L and IL-10 diffusion coefficient – D_N. We assume for the diffusion coefficient for cytokines the value of 1.25×10^{-3} cm^2 day^{-1}reported in Liao *et al.*[6], then $D_L = 1.45 \times 10^{-8}$ cm^2 s^{-1}, and $D_N = 1.45 \times 10^{-8}$ cm^2 s^{-1}.

Virus production coefficient – p. In Quirouette *et al.*[9] a production rate for influenza A virus in HRT of 8.4×10^{-6} $TCID_{50}$ mL^{-1} h^{-1} is reported, which is not suitable from a dimensional point of view. The parameter was then calibrated to reproduce the experimental results reported in Longhi *et al.*[17], Huang *et al.*[14], Zhou *et al.*[15] and Du *et al.*[16] as $p = 2.32 \times 10^{-5}$ s^{-1}.

Virus clearing coefficient – c. Values found in literature span from 0.6 day^{-1} = 6.94×10^{-6} s^{-1} for HIV replication in $CD4^+$ - T cell[13], to 0.22 h^{-1} = 6.1×10^{-5} s^{-1} for influenza A virus in HRT[9]. By

assimilation, we choose the value referred to HIV, then $c = 0.6$ day^{-1} = 0.6/86400 s^{-1} = 6.94×10^{-6} s^{-1}.

Infected CD4$^+$-T cells chemotactic coefficient – χ_I. To estimate this parameter we proceed in reverse, starting from the non-dimensional values estimated in Lai *et al*[10], where chemotactic coefficients spanning in the 10^{-3}÷10^{-2} non-dimensional range were analysed. We set as non-dimensional value for the coefficient the value 0.001, leading to a dimensional coefficient $\chi_I = 2 \times 10^{-16}$ cm^5 s^{-1} cell^{-1}.

Virus fugetactic coefficient – χ_V. As in the previous case, we set the non-dimensional value for the parameter to 0.05, leading to a dimensional coefficient $\chi_V = 5 \times 10^{-15}$ cm^5 s^{-1} cell^{-1}.

CD4$^+$-T cells decay rate – d_T. In Lai *et al.*[10] a non-dimensional value of 0.1 is given for CD4$^+$-T cells decay rate, which in our dimensional form should read as 10^{-5} s^{-1}. On the other hand, in Liao *et al.*[6] a decay rate for CD8$^+$-T cells of 3.47×10^{-6} s^{-1} has been estimated, whilst we set $d_T = 2 \times 10^{-6}$ s^{-1}.

CD4$^+$-T cells infection rate – k. In Quirouette *et al.*[9] an estimate for the infection rate of target cell by influenza A virus is given as 1.33×10^{-6} (TCID$_{50}$ mL^{-1})$^{-1}$ h^{-1}. In order to find a conversion from TCID$_{50}$ mL^{-1} h^{-1} to N$_{virus}$ cm^{-3} s^{-1}, as a working estimate we first adopt 1 TCID$_{50}$ mL^{-1} = 0.7 PFU mL^{-1}, and then 1 PFU = 1 number of infectious virus[12]. Hence,

$$k = \frac{1.33\times10^{-6}}{0.7} \times 2.78 \times 10^{-4} cm^3 s^{-1} cell^{-1} \approx 5.28 \times 10^{-10} cm^3 s^{-1} cell^{-1},$$

a value that turned out to be high for convergence purposes, while we calibrated the parameter as $k = 7.4 \times 10^{-15}$ cm^3 s^{-1} cell^{-1}.

CD4$^+$-T cells activation rate by IL-6 – ϕ_{21}, *T* cell inhibition rate by IL-10 – ϕ_{23}. In the absence of direct measures, we adopt by assimilation some parameter used in Liao *et al.*[6] for tumour cells, having regard to their size and magnitude. In particular, the death rate of tumour cells and the inhibition rate for tumour cells from IL-10, respectively 1.73×10^{-1} day^{-1} and 3.45×10^{-1} day^{-1}, give values spanning in the 2×10^{-6} s^{-1} ÷ 4×10^{-6} s^{-1} range. Therefore, we set $\phi_{21} = 2 \times 10^{-6}$ s^{-1} and $\phi_{23} = 4 \times 10^{-6}$ s^{-1}.

$CD4^+$-T cells production rate by M1 – ϕ_{22}, T cell inhibition rate by M2 – ϕ_{24}. These parameters where calibrated in order to match as close as possible the experimental findings in Huang *et al.*[14], Zhou *et al.*[15] and Du *et al.*[16], then we set $\phi_{22} = 1.67 \times 10^3$ s^{-1} and $\phi_{24} = 6.9$ s^{-1}.

Infected $CD4^+$-T cells decay rate – δ. We found in literature[13] that a decline rate for infected cells producing virus for HIV infected T cells is 0.39 day^{-1} = 4.5×10^{-6} s^{-1}, while in Lai *et al.*[10] a non-dimensional value of 0.2 for the same parameter is given. It follows that the decay rate for infected T cells can span in the 10^{-6} s^{-1} ÷ 10^{-5} s^{-1} range: we set $\delta = 2 \times 10^{-6}$ s^{-1}.

Infected $CD4^+$-T cells reduction rate due to hyper-inflammation – ϕ_{32}. This parameter has been calibrated in order to match as close as possible the experimental findings in Longhi *et al.*[17], Huang *et al.*[14], Zhou *et al.*[15] and Du *et al.*[16], then we set $\phi_{32} = 3.5 \times 10^{-13}$ cm^3 cell^{-1} s^{-1}.

IL-10 inhibition rate induced by IL-6 – ϕ_{73}. Nickaeen *et al.*[18] proposed an agent-based mathematical model for macrophage polarization, where the production/inhibition rates of several cytokines entering in their model have been estimated or harvested from literature. In particular, IL-12 in that model inhibits IL-4 production at a rate 9.26×10^{-5}, then by assimilating IL-12 to IL-6 and IL-4 to IL-10 we adopt such non-dimensional value which in dimensional form reads as $\phi_{73} = 9.26 \times 10^{-9}$ s^{-1}.

Nevertheless, it can be seen that all the parameters related to cytokine production/inhibition belong to the $9.26 \times 10^{-5} \div 2 \times 10^{-3}$ non-dimensional range, even if it comes in response to activation of several pathways that may or may not be involved in our case, but we trust that the orders of magnitude of the parameters are adaptable to our purposes. Similar estimates have been done in Frieboes *et al.*[19], for protein production rate and their ranges of variability, as well for protein decay rate, based on proteomic analysis, in a study aimed at the modelling of production, transport and shedding of proteins in a vascularized tumour. Hence, the remaining parameter estimate, is given accordingly, in order to match above mentioned experimental results[14-17]:

M1 macrophages production rate induced by IL-6 – ϕ_{41}. The non-dimensional estimate is 0.001, then $\phi_{41} = 2.4 \times 10^{-10}$ s^{-1}.

M1 macrophages inhibition rate induced by IL-10 – ϕ_{42}. The non-dimensional estimate is 0.0001, then $\phi_{42} = 2.4 \times 10^{-11}$ s^{-1}.

M2 macrophages promotion rate induced by IL-10 – ϕ_{51}. The non-dimensional estimate is 0.1, then $\phi_{51} = 2.4 \times 10^{-8}$ s^{-1}.

M2 macrophages inhibition rate induced by IL-6 – ϕ_{52}. The non-dimensional estimate is 0.001 and 0.002, then $\phi_{52} = 2.4 \times 10^{-10}$ s^{-1} and 4.8×10^{-10} s^{-1}.

IL-6 production rate induced by M1– ϕ_{61}. The non-dimensional estimate is 0.5, then $\phi_{61} = 2.08 \times 10^{-2}$ s^{-1}.

IL-6 inhibition rate induced by IL-10 – ϕ_{63}. The non-dimensional estimate is 0.01, then $\phi_{63} = 1 \times 10^{-6}$ s^{-1}.

IL-10 production rate induced by M2– ϕ_{71}. The non-dimensional estimate is 0.1, then $\phi_{71} = 4.15 \times 10^{-3}$ s^{-1}.

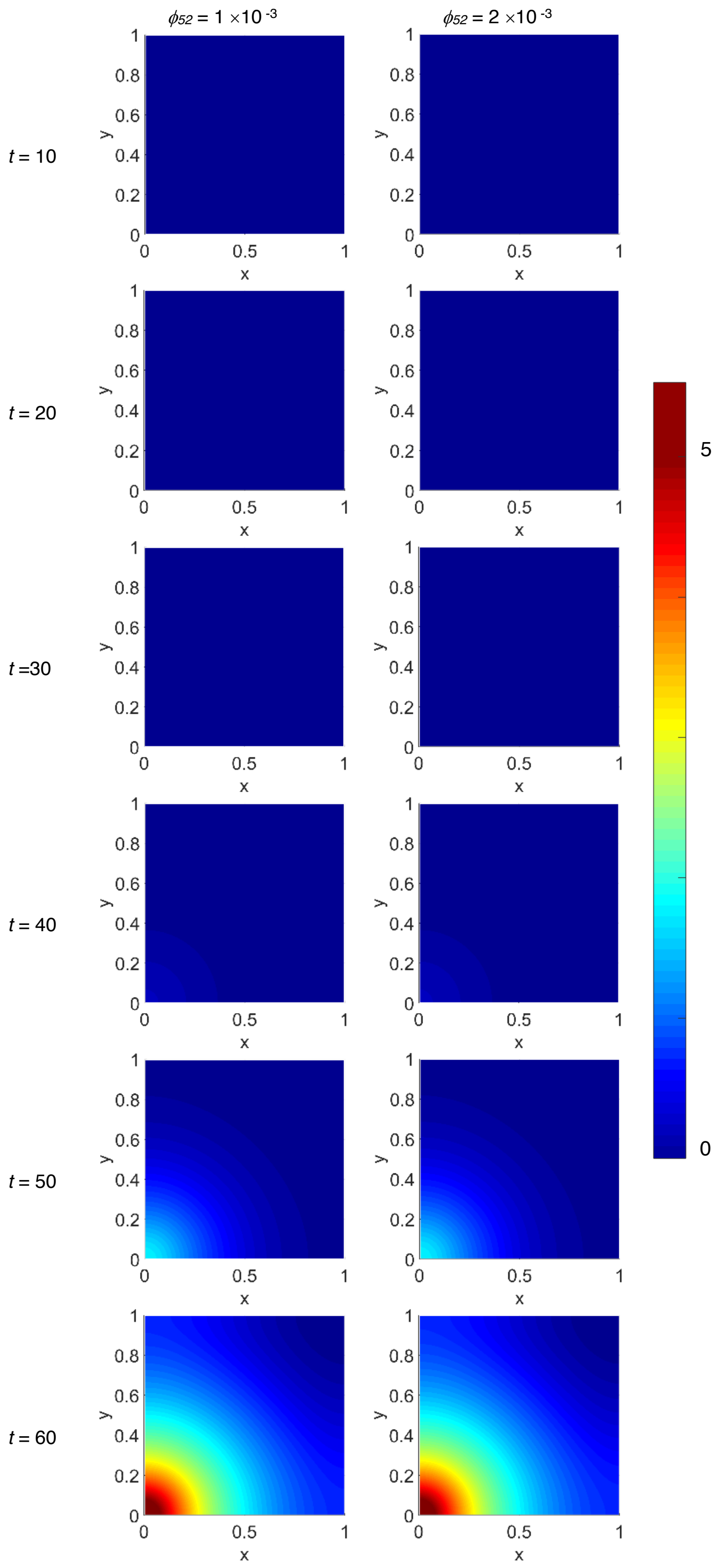

Supplementary Figure S1. Snapshots of the infected T cells density. The density is linearly mapped in colour scale between the blue and red colours in the [0,1]×[0,1] square domain. Starting from the top row, the variable evolution has been computed at t = 10 (~1.16 days), t = 20 (~2.3 days), t = 30 (~3.47 days), t = 40 (~4.63 days), t = 50 (~5.79 days) and t = 60 (~6.94 days), while the first and second column of panels refer to $\phi_{52} = 1 \times 10^{-3}$ and $\phi_{52} = 2 \times 10^{-3}$, respectively. All the other parameters are as in Table 1.

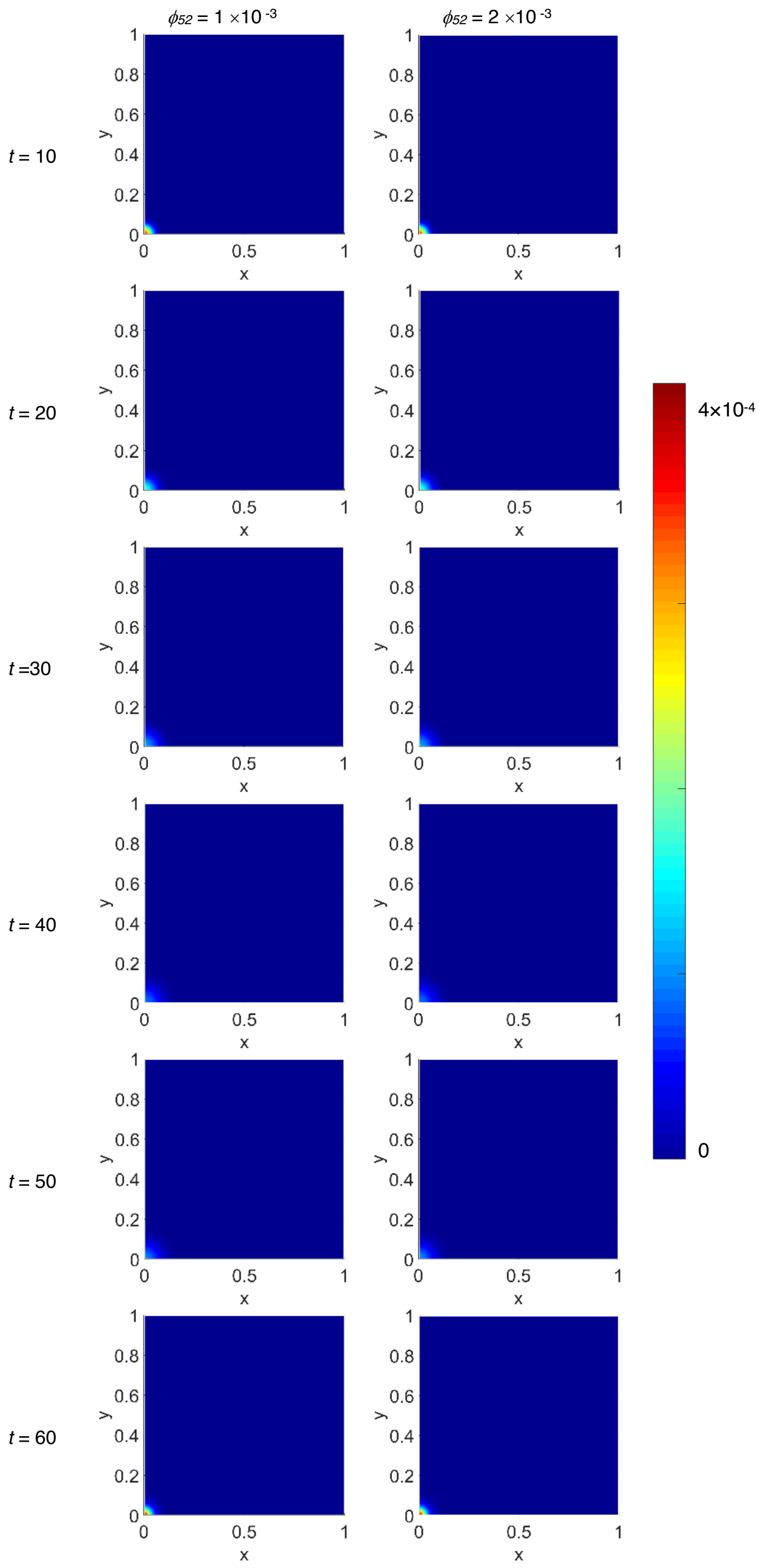

Supplementary Figure S2. Snapshots of the M1 cells density. The density is linearly mapped in colour scale between the blue and red colours in the [0,1]×[0,1] square domain. Starting from the top row, the variable evolution has been computed at t = 10 (~1.16 days), t = 20 (~2.3 days), t = 30 (~3.47 days), t = 40 (~4.63 days), t = 50 (~5.79 days) and t = 60 (~6.94 days), while the first and second column of panels refer to $\phi_{52} = 1 \times 10^{-3}$ and $\phi_{52} = 2 \times 10^{-3}$, respectively. All the other parameters are as in Table 1.

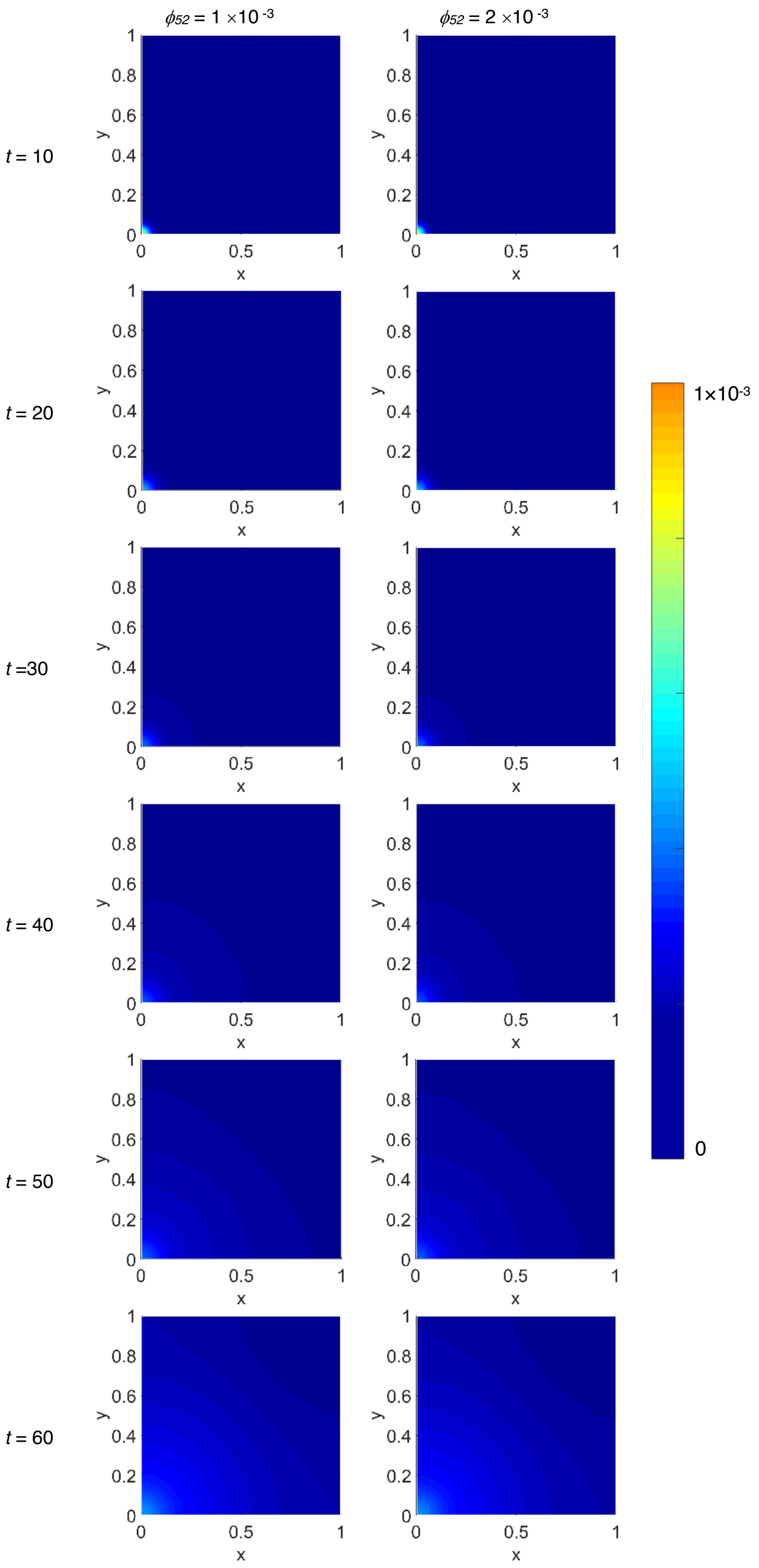

Supplementary Figure S3. Snapshots of the M2 cells density. The density is linearly mapped in colour scale between the blue and red colours in the [0,1]×[0,1] square domain. Starting from the top row, the variable evolution has been computed at t = 10 (~1.16 days), t = 20 (~2.3 days), t = 30 (~3.47 days), t = 40 (~4.63 days), t = 50 (~5.79 days) and t = 60 (~6.94 days), while the first and second column of panels refer to $\phi_{52} = 1 \times 10^{-3}$ and $\phi_{52} = 2 \times 10^{-3}$, respectively. All the other parameters are as in Table 1.

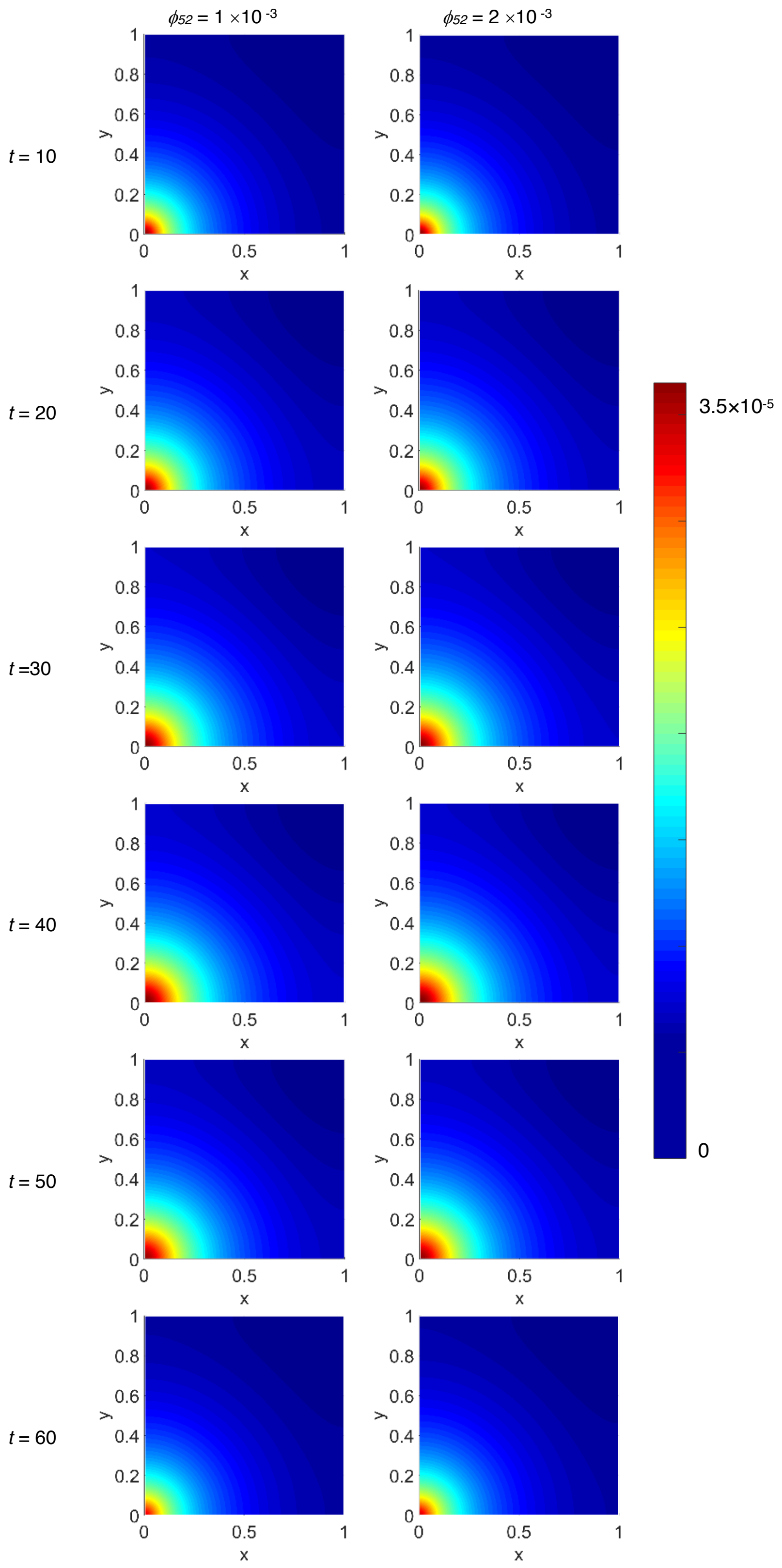

Supplementary Figure S4. Snapshots of the IL-6 density. The density is linearly mapped in colour scale between the blue and red colours in the [0,1]×[0,1] square domain. Starting from the top row, the variable evolution has been computed at t = 10 (~1.16 days), t = 20 (~2.3 days), t = 30 (~3.47 days), t = 40 (~4.63 days), t = 50 (~5.79 days) and t = 60 (~6.94 days), while the first and second column of panels refer to ϕ_{52} = 1×10^{-3} and $\phi_{52} = 2 \times 10^{-3}$, respectively. All the other parameters are as in Table 1

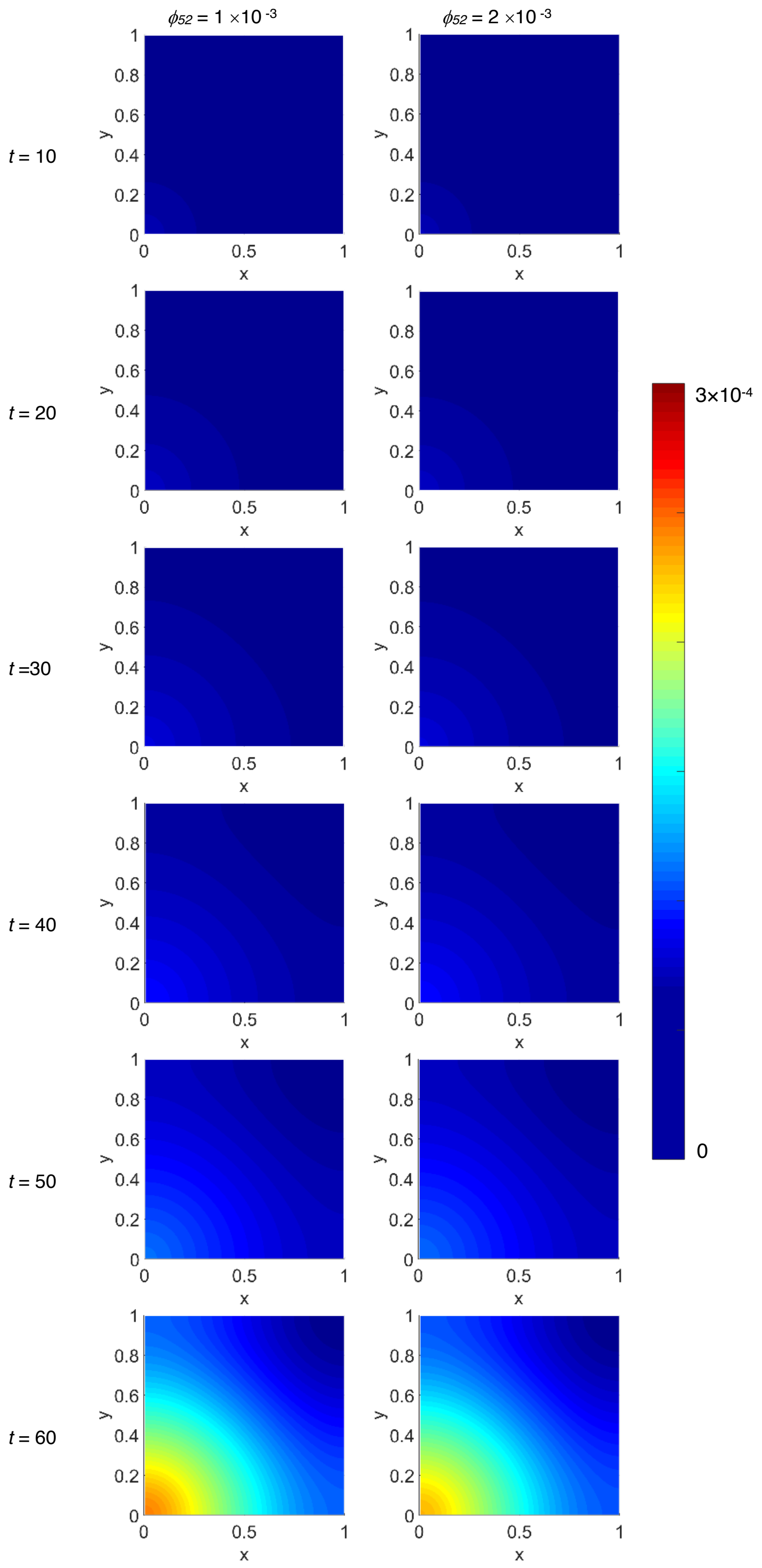

Supplementary Figure S5. Snapshots of the IL-10 density. The density is linearly mapped in colour scale between the blue and red colours in the [0,1]×[0,1] square domain. Starting from the top row, the variable evolution has been computed at t = 10 (~1.16 days), t = 20 (~2.3 days), t = 30 (~3.47 days), t = 40 (~4.63 days), t = 50 (~5.79 days) and t = 60 (~6.94 days), while the first and second column of panels refer to $\phi_{52} = 1 \times 10^{-3}$ and $\phi_{52} = 2 \times 10^{-3}$, respectively. All the other parameters are as in Table 1.

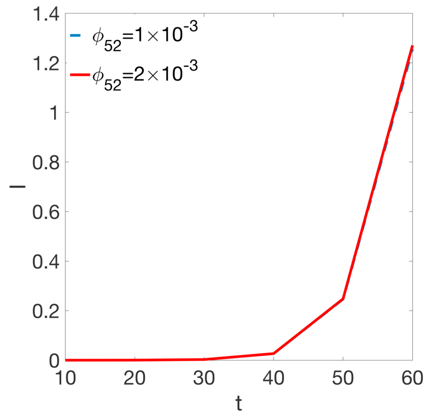

Supplementary Figure S6. Calculated amount of infected T cells present in the domain at each time step. Points in each curve have been obtained by numerical integration over the spatial domain of the maps shown in Supplementary Figs. S1 online.

References

1. Amoddeo, A. Modelling avascular tumor growth: approach with an adaptive grid numerical technique. *J. Multiscale Model.* **9,** 1840002; doi: 10.1142/S1756973718400024 (2018).
2. Amoddeo, A. A moving mesh study for diffusion induced effects in avascular tumour growth. *Comput. Math. Appl.* **75,** 2508-2519; https://doi.org/10.1016/j.camwa.2017.12.024 (2018).
3. Amoddeo, A. Nematodynamics modelling under extreme mechanical and electric stresses. *J. Phys. Conf. Ser.* **574,** 012102; doi:10.1088/1742-6596/574/1/012102 (2015).
4. Sender, R. *et al.* The total number and mass of SARS-CoV-2 virions. *PNAS* **118,** e2024815118; https://doi.org/10.1073/pnas.2024815118 (2021).
5. Leonard, F. *et al.* Enhanced performance of macrophage-encapsulated nanoparticle albumin-bound-paclitaxel in hypo-perfused cancer lesions. *Nanoscale* **8,** 12544–12552; doi:10.1039/c5nr07796f (2016).
6. Liao, K.L., Bai, X.F. & Friedman A. Mathematical modeling of interleukin-27 induction of anti-tumor T cells response. *PLoS One* **9,** e91844; doi:10.1371/journal.pone.0091844 (2014).
7. https://www.invivogen.com/sites/default/files/invivogen/products/files/rhil_6_tds.pdf
8. https://www.rndsystems.com/resources/articles/interleukin-10-il-10-family
9. Quirouette, C., Younis, N.P., Reddy, M.B. & Beauchemin, C.A.A. A mathematical model describing the localization and spread of influenza A virus infection within the human respiratory tract. *PLoS Comput. Biol.* **16,** e1007705; https://doi.org/10.1371/journal.pcbi.1007705
10. Lai, X. & Zou X. A reaction diffusion system modeling virus dynamics and CTL response with chemotaxis. *Disc. Cont. Dyn. Syst. B* **21,** 2567-2585; doi: 10.3934/dcdsb.2016061 (2016).
11. Owen, M.R., Byrne, H.M. & Lewis, C.E. Mathematical modelling of the use of macrophages as vehicles for drug delivery to hypoxic tumour sites. *J. Theor. Biol.* **226,** 377–391; doi:10.1016/j.jtbi.2003.09.004 (2004).
12. https://molecularepi.com/atccv30no1.pdf
13. Zitzmann, C. & Kaderali L. Mathematical analysis of viral replication dynamics and antiviral treatment strategies: from basic models to age-based multi-scale modeling. *Front. Microbiol.* **9,** 1546; doi: 10.3389/fmicb.2018.01546 (2018).
14. Huang, C. *et al.* Clinical features of patients infected with 2019 novel coronavirus in Wuhan, China. *Lancet* **395,** 497–506; https://doi.org/10.1016/S0140-6736(20)30183-5 (2020).

15. Zhou, F. *et al.* Clinical course and risk factors for mortality of adult inpatients with COVID-19 in Wuhan, China: a retrospective cohort study. *Lancet* **395,** 1054–62; https://doi.org/10.1016/S0140-6736(20)30566-3 (2020).
16. Du, Y. *et al.* Cannabinoid 2 receptor attenuates inflammation during skin wound healing by inhibiting M1 macrophages rather than activating M2 macrophages. *J. Inflamm.* **15,** 25; https://doi.org/10.1186/s12950-018-0201-z (2018).
17. Longhi, M.P. *et al.* Interleukin-6 is crucial for recall of influenza-specific memory $CD4^+$ T cells. *PLoS Pathog.* **4,** e1000006; doi:10.1371/journal.ppat.1000006 (2008).
18. Nickaeen, N., Ghaisari, J., Heiner, M., Moein, S. & Gheisari Y. Agent-based modeling and bifurcation analysis reveal mechanisms of macrophage polarization and phenotype pattern distribution. *Sci. Rep.* **9,** 12764; https://doi.org/10.1038/s41598-019-48865-z (2019).
19. Frieboes, H.B., Curtis, L.T., Wu, M., Kani, K. & Mallick, P. Simulation of the protein-shedding kinetics of a fully vascularized tumor. *Cancer Inform.***14,** 163–175; doi: 10.4137/CIN.S35374 (2015).
